# Proteome profiling reveals opportunities to investigate biomarkers of oxidative stress and immune responses in blubber biopsies from free-ranging baleen whales

**DOI:** 10.1093/conphys/coae059

**Published:** 2024-08-19

**Authors:** Joanna Kershaw, Christian Ramp, Richard Sears, Ailsa Hall, Davina Derous

**Affiliations:** Sea Mammal Research Unit, Scottish Oceans Institute, University of St Andrews, St Andrews, KY16 8LB; School of Biological Sciences, University of Aberdeen, Aberdeen, UK; Mingan Island Cetacean Study, Saint Lambert, Québec, Canada; Sea Mammal Research Unit, Scottish Oceans Institute, University of St Andrews, St Andrews, KY16 8LB; Mingan Island Cetacean Study, Saint Lambert, Québec, Canada; Mingan Island Cetacean Study, Saint Lambert, Québec, Canada; Sea Mammal Research Unit, Scottish Oceans Institute, University of St Andrews, St Andrews, KY16 8LB; School of Biological Sciences, University of Aberdeen, Aberdeen, UK

**Keywords:** Cetaceans, proteomics, bioinformatics, health, blubber

## Abstract

Over 25% of cetacean species worldwide are listed as critically endangered, endangered or vulnerable by the International Union for Conservation of Nature. Objective and widely applicable tools to assess cetacean health are therefore vital for population monitoring and to inform conservation initiatives. Novel blubber biomarkers of physiological state are examples of such tools that could be used to assess overall health. Proteins extracted from blubber likely originate from both the circulation and various cell types within the tissue itself, and their expression is responsive to signals originating from other organs and the nervous system. Blubber proteins can therefore capture information on physiological stressors experienced by individuals at the time of sampling. For the first time, we assess the feasibility of applying shotgun proteomics to blubber biopsy samples collected from free-ranging baleen whales. Samples were collected from minke whales (*Balaenoptera acutorostrata*) (*n* = 10) in the Gulf of St Lawrence, Canada. Total protein was extracted using a RIPA cell lysis and extraction buffer-based protocol. Extracted proteins were separated and identified using nanoflow Liquid Chromatography Electrospray Ionization in tandem with Mass Spectrometry. We mapped proteins to known biological pathways and determined whether they were significantly enriched based on the proteome profile. A pathway enrichment map was created to visualize overlap in tissue-level biological processes. Amongst the most significantly enriched biological pathways were those involved in immune system function: inflammatory responses, leukocyte-mediated immunity and the humoral immune response. Pathways associated with responses to oxidative stress were also enriched. Using a suite of such protein biomarkers has the potential to better assess the overall health and physiological state of live individuals through remote biopsy sampling. This information is vital for population health assessments to predict population trajectories, and ultimately guide and monitor conservation priorities and initiatives.

## Teaser

Widely applicable tools to assess cetacean health are vital for informing conservation priorities. However, logistical constraints mean that assessing the health of free-ranging baleen whales is challenging. Here, we identify a suite of protein biomarkers in blubber biopsy samples from live individuals as potential indicators of health and physiological state.

## Introduction

Wildlife research is increasingly using molecular methods for monitoring biodiversity and guiding conservation initiatives (Hoban *et al.*, 2022; Tiwari and Rajwanshi, 2022). Specifically, integrating genomics, transcriptomics, proteomics and metabolomics approaches will help researchers understand and measure individual animal health as well as biological diversity and ecosystem health, with the aim of ultimately enabling policy makers to translate multi-omics results into practical solutions to conserve biodiversity. ‘Omics approaches are becoming increasingly accessible, and proteomics in particular is expected to advance rapidly with the emergence of spatial proteomics as a new tool. These technological advancements and the increasing accessibility of sequenced genomes enable critical research to provide valuable new insights in non-model organisms (Heck and Neely, 2020). Data generated by high-throughput technologies across a range of species, tissue types and time scales therefore represent huge opportunities for improving our understanding of wildlife health, and by proxy, the health of their environments. By taking advantage of the methods developed for fundamental research in model study systems, and terrestrial species, we can now apply these invaluable ‘omics approaches to marine science. This will allow us to develop new tools to assess the health of individuals and populations in marine ecosystems at a level previously unavailable. Using these approaches to develop long-term temporal and spatial monitoring programmes of marine top predator health, as sentinels of their environment that are increasingly impacted by climate change and anthropogenic stressors (Schwacke *et al.*, 2013; Hazen *et al.*, 2019; Bestley *et al.*, 2020; Williamson *et al.*, 2021), will allow the broad integration of valuable molecular data into marine conservation.

Obtaining samples from live, free-ranging animals remains logistically and ethically challenging. Yet, information from such tissue samples can provide essential information for the management and conservation of the species. To date, ‘omics approaches in marine mammals to assess stress state and overall health include only a handful of species. In cetaceans, transcriptomic analyses of skin in bottlenose dolphins (*Tursiops truncatus*) have been used to identify potential contaminant-responsive markers involved in cellular architecture, immune response and oxidative stress (Neely *et al.*, 2018), and gene expression variability associated with sex, pregnancy status and geographic location (Trego *et al.*, 2019). In bowhead whales (*Balaena mysticetus*) and beluga (*Delphinapterus leucas*), investigations to understand variation in the metabolic function of cetacean blubber showed that genes encoding important adipokines and lipases were expressed more highly in inner compared to outer blubber (Ball *et al.*, 2017). In pinnipeds, fasting adaptations, hypoxic stress tolerance and responses to chronic stress have been investigated using transcriptomic analyses of the muscle and blubber tissue of northern elephant seals (*Mirounga angustirostris*) (Khudyakov *et al.*, 2015; Martinez *et al.*, 2018; Deyarmin *et al.*, 2019). Assessments of gene expression variation through blubber tissue depth in this species have also produced insights into the functional stratification of blubber layers (Khudyakov *et al.*, 2022a). Together, these non-targeted and system-wide approaches have provided valuable information on the molecular pathways regulating adaptations such as fasting, hypoxia and environmental stress responses in marine mammals.

Proteomics approaches have similarly been applied to elephant seal blubber samples to investigate the impacts of repeated stressors on blubber tissue function and understand what this could mean in terms of altering the metabolism of vital energy stores of individuals (Deyarmin *et al.*, 2020). Proteins involved in energy and tissue homeostasis were affected, and it was suggested that these could be used as potential markers of repeated stress in marine mammals (Deyarmin *et al.*, 2020). Importantly, for species from which blubber biopsies but not blood samples can be regularly obtained, quantification of such proteins could potentially be used to assess chronic stress. Quantifying protein expression is therefore valuable in cetacean research whereby remotely obtained, shallow-depth blubber biopsies can be obtained from a range of species (Noren and Mocklin, 2012; Hunt *et al.*, 2013). To date, untargeted proteomic analysis of cetacean blubber has only been applied to samples obtained from stranded cetaceans (Kershaw *et al.*, 2018), as studies in live animals have focused on lipidomic (Bories *et al.*, 2021) or metabolomic (Simond *et al.*, 2020) markers. The power of proteomic investigations is that these proteins are likely derived from both the adipocytes themselves, as well as from circulation. They can therefore provide insight into the regulation of biological processes and the production of signalling proteins to capture information on physiological stresses experienced from a cellular to a whole-organism level at the time of sampling. Here, remotely obtained, shallow, biopsy samples were collected from live, free-ranging minke whales (*Balaenoptera acutorostrata*) during their summer feeding season in the Gulf of St Lawrence, Canada. These samples were processed to assess the feasibility of applying proteomic methods to superficial blubber tissue samples obtained from live animals and stored under field conditions, with the aim of assessing firstly, the range of proteins present in the tissue, and secondly, the biological processes that they are involved in. These results ultimately provide insight into the potential for the use of proteomic methods for more targeted investigations into live animal health.

## Materials and Methods

### Biopsy sample collection

For this proof-of-concept study, biopsy samples were collected from 10 minke whales by the Mingan Island Cetacean Study in Quebec, Canada in 2013. Sampling occurred during the summer feeding season, from June to September in the Jacques Cartier Passage of the Gulf of St. Lawrence (49° 36’ N, 64° 20’ W). Biopsies were collected from rigid-hulled, inflatable boats using a crossbow and hollow-tipped (40 mm in length and 8 mm in diameter, Finn Larsen (CETA-dart, DK)) arrow system from the dorsal and flank areas of the individuals. All samples were stored in glass vials and on ice immediately after collection for between 3 and 6 h, and subsequently frozen at −20°C until proteomic analysis in 2018. These animals were sexed genetically using the skin (Palsbøll *et al.*, 1992), and were all females. Minke whales are thought to reproduce annually (Lockyer, 1984; Horwood, 1989), so the individuals sampled here are likely a mixture of both pregnant and non-pregnant females.

### Total protein extraction, quantification and visualization

Biopsies were on average 2.19 ± 0.67 cm in length. Full-length, longitudinal subsamples of the biopsies (0.1 ± 0.01 g) were taken, and total protein was extracted using RIPA lysis and extraction buffer, quantified and visualized as previously described (Kershaw *et al.*, 2018). Briefly, the frozen tissue was placed on ice with RIPA lysis and extraction buffer (Thermo Fisher Scientific) containing 2× concentration of protease inhibitors (Pierce Protease Inhibitor Mini Tablets). The samples were homogenized and replaced on ice, centrifuged and the protein-containing infranatant collected. This protein fraction was centrifuged, replaced on ice, the infranatant collected and used for analysis. A Pierce™ 660-nm Protein Assay (22 662, Thermo Scientific, Rockford, USA) was used to quantify the total protein content in the extracts. Each extract was assayed in duplicate, with four extracts assayed in replicate on the same plate, with an intra-assay coefficient of variance (CV) of <10% considered acceptable. All extracts were assayed on a second plate with an inter-assay CV of <15% considered acceptable. Intra- and inter-assay CVs were 5.62 and 10.48%, respectively. Proteins were then separated and visualized using standard 4–12% NuPAGE 1D Bis-Tris mini gel running, staining, de-staining and imaging processes as previously described (Kershaw *et al.*, 2018; Kershaw *et al.*, 2019). Proteins were separated using 1D gels prior to protease digestion for proteomic analyses as this method can rapidly clean up low protein content samples with minimal losses (Goldman *et al.*, 2019).

### Protein identification

A total of 11 protein bands were excised from four of the blubber extracts run on the same 1D SDS-PAGE gel. In order to potentially capture the greatest range in proteins present, bands from individuals sampled early in the feeding season (June), the middle of the feeding season (July and August) and late in the feeding season (September) were used for analysis. The excised bands covered the full size range of separated proteins from the largest ones of >200 kDa, down to the smallest bands visible at ~10 kDa. Bands were stored in individual low protein-binding microcentrifuge tubes at 4–8°C before subsequent protein identification. Bands were analysed using nanoflow Liquid Chromatography Electrospray Ionization in tandem with Mass Spectrometry (nLC-ESI MS/MS) of in-gel trypsin digests.

As previously described, the excised gel band was cut into 1-mm cubes (Kershaw *et al.*, 2018). These were then subjected to in-gel digestion, using a ProGest Investigator in-gel digestion robot (Genomic Solutions, Ann Arbor, MI) using standard protocols (Shevchenko et al., 1996). Briefly, the gel cubes were de-stained by washing with acetonitrile and subjected to reduction and alkylation before digestion with trypsin at 37°C. The peptides were extracted with 10% formic acid and concentrated down 20× using a SpeedVac (ThermoSavant). The peptides were then injected on an Acclaim PepMap 100 C18 trap and an Acclaim PepMap RSLC C18 column (ThermoFisher Scientific) using a nanoLC Ultra 2D plus loading pump and nanoLC as-2 autosampler (Eksigent). The peptides were eluted with a gradient of increasing acetonitrile, containing 0.1% formic acid (5–40% acetonitrile in 6 min, 40–95% in a further 2.5 min, followed by 95% acetonitrile to clean the column, before re-equilibration to 5% acetonitrile). The eluate was sprayed into a TripleTOF 5600+ electrospray tandem mass spectrometer (Sciex, Foster City, CA) and analysed in Information-Dependent Acquisition (IDA) mode, performing 250 ms of MS followed by 100 ms MS/MS analyses on the 20 most intense peaks seen by MS. The MS/MS data file generated via the ‘Create mgf file’ script in PeakView (Sciex) was analysed using the Mascot search algorithm (Matrix Science), against the NCBInr database with no species restriction (65 519 838 to 93 482 448 sequences), trypsin as the cleavage enzyme and carbamidomethyl as a fixed modification of cysteines and methionine oxidation as a variable modification. The peptide mass tolerance was set to 20 ppm and the MS/MS mass tolerance to ±0.05 Da.

A protein was accepted as identified if it had two or more peptides with Mascot Ion Scores above the Identity Threshold (*P* < 0.05) and, for those proteins identified by only two peptides, the MS/MS spectral assignments fulfil the criteria as previously described (Jonscher, 2005). The sequences matched homologous vertebrate proteins.

### Biological processes enrichment analysis

Following protein identification, protein names were uploaded to ClueGO v2.5.10, a plug-in for Cytoscape (v3.10.0), an open-source software platform for visualizing complex networks in molecular and systems biology, genomics and proteomics (Bindea *et al.*, 2009). *Homo sapiens* was selected as the reference species. Identifier was set to ‘Symbol ID’ and the data set ‘GO_BiologicalProcess-EBI-UniProt-GOA-ACAP-ARAP_05.07.2023’ was used to map the identified proteins to the biological processes that they are involved in. The ‘Enrichment/Depletion’ default statistical test setting was used (two-sided hypergeometric test) with an adjusted *P*-value cut-off set to 0.05 (Bonferroni step-down corrected). Gene Ontology (GO) tree levels were specified between minimum level 0 and maximum level 3. After *P*-value significance selection criteria (<0.05) were applied, 125 GO:BP were significantly enriched. The number of proteins mapped to each GO:BP was calculated, and the proportion of the GO:BP represented by these proteins was assessed.

For visualization purposes, a network was constructed of the significantly enriched GO:BP. Any GO:BP that had >50.0% overlap in proteins were merged to produce a final 32 functional groups. Kappa Score Threshold as a measure of network connectivity was set to 0.4 and connects the GO:BP with a line. These functional groups are then coloured individually and named based on the highest significance of the GO:BP in that group.

**Figure 1:**
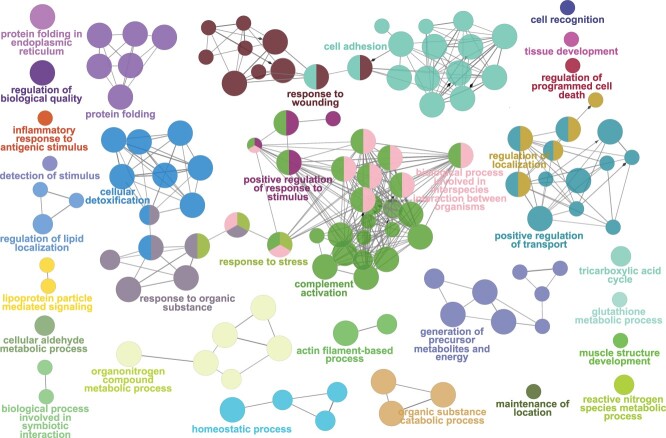
Significantly enriched GO:BP in the blubber extracts. Each point represents a biological process, and the link between them indicates a Kappa Score of >0.4 (similarity between the proteins in the GO:BP). Biological processes are coloured according to functionally grouped terms automatically identified by ClueGO. The most significant GO:BP term of a group is highlighted in the network. A biological process can be a member of more than one, larger group processes and can have thus more than one colour.

## Results

### Protein yield and identification

The total protein yield from the 10 biopsy extracts ranged from 971.17 to 7010.80 ng/g with an average of 3893.95 ± 1602.70 ng/g. A total of 434 unique proteins were identified across the 11 gel bands extracted. Almost half (48.8%) of the proteins identified were only detected once, and just <20% were detected more than five times across these extracts. The six most frequently identified proteins were haemoglobin, fatty acid-binding protein, apolipoproteins, immunoglobulins, serum albumin and perilipins.

### Biological processes enrichment analysis

**Figure 2:**
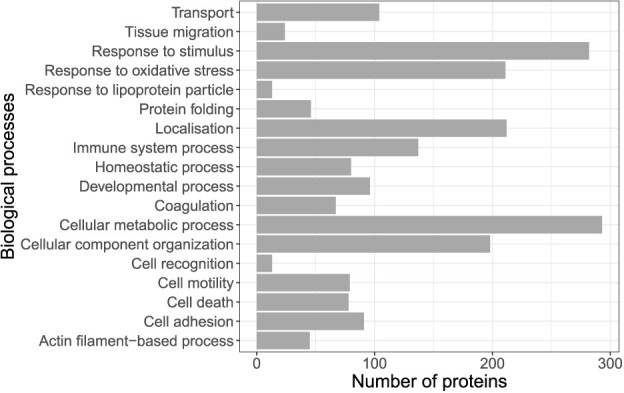
Number of proteins in each of the ancestor GO:BP of the significantly enriched GO:BP in blubber as determined by Quick GO (https://www.ebi.ac.uk/QuickGO/).

In total, 125 GO:Biological Processes (BP) were significantly enriched in blubber (Fig. 1, Supplementary Table S1). These 125 GO:BP were manually added to broader terms based on the Ancestor chart of GO using QuickGO, and the number of proteins were noted for each of these ancestor GO terms (Fig. 2). We then constructed the similarity network of these 125 GO:BP, and after merging GO:BP terms with >50% similarity in proteins, 32 functional groups were automatically identified by ClueGO (Fig. 1, Supplementary Table S1). Proportionally, the largest group of enriched biological processes was involved in ‘cellular metabolic processes’, which included proteins involved in small molecule biosynthetic processes, the regulation of lipid localization, fatty acid metabolism and adipogenesis, for example. The second largest group of proteins were those involved in ‘response to stimulus’ and included proteins involved in detoxification mechanisms, as well as alpha-2-macroglobulin for example, involved in disrupting inflammatory cascades. The third largest group were proteins involved in ‘responses to oxidative stress’, including heat shock proteins (HSPs) 28, 27, 70, 90 and 84. Given that HSPs have multiple other functions including important roles as molecular chaperones, they belong to other GO categories as well.

Additionally, proteins involved in ‘localization’ made up a large proportion of important proteins that included any process that activates or increases the frequency, rate or extent of the directed movement of substances (such as macromolecules, small molecules, ions) into, out of, within or between cells. These included numerous biomolecule transport proteins including ceruloplasmin, vitamin D-binding protein and serotransferrin. A wide range of immune system proteins were present, involved in inflammatory responses, leukocyte-mediated immunity and the humoral immune response. Complement activation processes were identified that form part of the activation of the complement cascade allowing for the direct killing of microbes, the disposal of immune complexes and the regulation of various other immune processes. Specifically, a number of complement proteins were identified; C3, C4, C5, C6, C7, C8 and C9. Processes involved in ‘wound healing’ consisted of any process that modulates the rate, frequency or extent of the series of events that restore integrity to a damaged tissue following an injury. This group included proteins such as annexins, coagulation factors and plasminogens. Finally, proteins involved in cell adhesion included those responsible for the attachment of one cell to another via adhesion molecules including integrins, cadherins and selectins.

## Discussion

For the first time, a shotgun proteomic approach was successfully applied to blubber biopsy samples obtained from free-ranging baleen whales. The 434 unique proteins identified in minke whale blubber tissue here are comparable to the total 564 unique proteins identified in elephant seal blubber (Deyarmin *et al.*, 2020). These biopsy samples were taken over a 4-month period (June to September, inclusive) of the summer feeding season where minke whales, like other mysticetes, are expected to fatten considerably (Christiansen *et al.*, 2013). As such, the individuals were in varying states of lipolysis and lipogenesis at the time of sampling, therefore providing a valuable snapshot of the range of tissue processes, including adipogenesis and tissue remodelling that were taking place at various stages over the feeding season.

### Biological processes identified: implications for specific biomarker targets

As expected, over half of the identified proteins were involved in cellular metabolism. This is in keeping with previous transcriptomic and proteomic work on elephant seal blubber, showing that metabolism was the most significantly enriched biological process (Khudyakov *et al.*, 2017; Deyarmin *et al.*, 2020). For example, the identification of adiponectin here, an adipokine involved in a range of physiological processes including lipid metabolism, energy regulation, immune response and inflammation (Khoramipour *et al.*, 2021), is especially of interest for biomarker development given its role in fat metabolism and whole-body homeostasis in other mammals. Overall, these results support the growing understanding that marine mammal blubber, like other adipose tissue types, is a metabolically active organ that is not only the primary storage site for excess energy, but is also involved in the regulation of metabolic homeostasis both at a tissue and whole-organism level.

The identification of a suite of proteins involved in lipid droplet homeostasis, fatty acid metabolism, lipid transport, lipolysis and adipogenesis showed the dynamic nature of lipid metabolism processes in blubber. Apolipoproteins were abundant and play important roles in the synthesis and catabolism of plasma lipoproteins, lipid transport and the activation of various enzymes involved in lipid and lipoprotein metabolism (Dominiczak and Caslake 2011; Liu *et al.*, 2021). Similarly, apolipoproteins dominated the suite of proteins that responded to fasting in northern elephant seals (Khudyakov *et al.*, 2022b), and were thought to contribute to the suppression of lipogenesis and maintenance of high levels of lipid mobilization during fasting. Fatty acid-binding proteins were also identified, some of which are involved in intracellular trafficking and targeting of fatty acids and modulating lipolytic rate (Frühbeck *et al.*, 2001), but are sensitive to metabolic or inflammatory stress (Makowski and Hotamisligil, 2004). Quantification of such proteins and the ratios between them could be a useful avenue of future research to understand the regulation of feeding- and fasting-related changes in blubber throughout the seasonal cycles of baleen whales.

Proteins involved in tissue responses to oxidative stress, defined as a disruption in the balance between the production of reactive oxygen species and anti-oxidant defences (Betteridge, 2000), were also significantly enriched. Specifically, six different HSPs were identified in the blubber extracts, which are known to mitigate against oxidative and proteotoxic stress through a combination of their roles as molecular chaperones and as anti-oxidants (Feder and Hofmann, 1999). In grey seals (*Halichoerus grypus*), greater requirements for HSPs and other anti-oxidants have been hypothesized to be the result of rapid protein synthesis and high metabolic fuel availability during juvenile growth (Bennett *et al.*, 2014). During the lactation fast of capital breeding female grey seals, another energetically demanding period of their life cycle, trade-offs occur between reproduction and somatic maintenance (i.e. anti-oxidative stress mechanisms). In these lactating females, there is greater oxidative damage in blubber tissues whilst cellular defences are lowered compared to actively foraging females (Armstrong *et al.*, 2023). It was concluded that these data are therefore in keeping with the ‘life history oxidative stress theory’, which predicts that allocation of resources to cellular maintenance, protection and repair, such as the production of HSPs, is lowered during resource-limited but energetically demanding periods (e.g. lactation in capital breeders), which ultimately results in greater oxidative damage (Armstrong *et al.*, 2023). Baleen whale species experience similar fasting and energetically demanding periods in their life cycle during lactation and migration, and thus understanding the role of cellular stress and anti-oxidant defences in blubber is vital for understanding baleen whale health. Future work aiming to assess the trade-offs between the production of HSPs and other anti-oxidant cellular defences, with cellular damage, protein carbonylation and lipid peroxidation for example, could provide valuable biomarker information regarding the fasting state and potential resource limitation of baleen whales on their feeding grounds.

Lastly, a range of proteins involved in immune system function was identified. The largest functional group were proteins involved in the complement pathway, and thus part of the innate immune response. Adipocytes are known to synthesize proteins involved in the activation of the alternative complement pathway (Shim *et al.*, 2020), which was originally viewed as a first line of defence against microbial infections. However, new research suggests wider functions of the complement system that extend beyond these defensive roles and involve a diverse range of processes including clearance of immune complexes, complementing lymphocyte functions, tissue regeneration and metabolism (Shim *et al.*, 2020). Here, other circulating immune system proteins involved in inflammation included haptoglobin and transferrin. Immunoglobulins and annexins were also identified, further supporting recent evidence of the presence of transcriptionally active cells involved in immune system function in marine mammal blubber (Khudyakov *et al.*, 2022a). Proteins involved in platelet activation, response to wounding and coagulation were also identified, in keeping with recent work on elephant seals that demonstrated that the outermost blubber layer had a high proportion of CD4+ immune cells, suggesting higher capacity for response to tissue injury (Khudyakov *et al.*, 2022a). Additionally, wound healing with blubber tissue remodelling in dolphins is characterized by high levels of inflammatory cell infiltration (Su *et al.*, 2022). Thus, both innate and adaptive components of the immune system were present in the tissue, and future work to isolate and quantify such proteins or quantify mRNA transcripts could provide information regarding wound healing and tissue repair (from predation attempts or anthropogenic injury, for example), as well as immune system function and response (as a result of infectious disease, for example). Together, these could provide candidate biomarkers for individual animal health and physiological state assessments.

### Insights into blubber function and tissue perfusion

The functional, molecular and morphological differences between marine mammal blubber and subcutaneous adipose tissue of other mammals are not yet fully understood. Increasing numbers of ‘omics studies across species are demonstrating the complexity of blubber function and providing insight into the importance of blubber metabolism not only in terms of responding to short- and longer term energetic demands, but to whole-body homeostasis and individual health (Khudyakov *et al.*, 2015; Kershaw *et al.*, 2018; Martinez *et al.*, 2018; Deyarmin *et al.*, 2019). Expanding our understanding of cell types within the tissue to show a heterogeneous mix of white adipocytes, brown adipocytes, connective tissue, muscle and nerve fibres and immune cells (Hashimoto *et al.*, 2015; Khudyakov *et al.*, 2022a) has been invaluable for our interpretations of the proteins secreted by both the adipocyte and non-adipocyte tissue fraction. Additionally, as stratification through blubber depth is known to impact morphological structure (Gómez-Campos *et al.*, 2015), fat content and components (Koopman, 2007) and molecular profiles (Khudyakov *et al.*, 2022a), protein content and composition are also likely to vary between superficial and deeper layers and warrants investigation.

The proteins identified here are typical of adipose tissue, cutaneous tissue and those present in circulation. Therefore, proteins identified here are likely a mixture of blubber proteins together with plasma proteins, and as such, blubber tissue sampling can provide information on both the proteins produced and metabolized in the tissue itself as well as those in circulation. Histological analyses of cetacean blubber tissue (McClelland *et al.*, 2012) as well as non-invasive measures of blubber perfusion in seals (McKnight *et al.*, 2019; Oller *et al.*, 2021) show that blubber is highly vascularized with periodic fluctuations in blood flow over minutes and hours. Therefore, for most cetacean species where routine blood sampling is either impossible or highly impractical, blubber sampling may provide insight into short-term changes in circulating patterns of biomarkers of interest if interpreted carefully considering the short-term peripheral vasoconstriction that occurs as part of the dive response (McKnight *et al.*, 2019). Additionally, the identification of extra-cellular matrix proteins could also provide important information on physiological state given these non-cellular components are known to provide crucial biochemical and biomechanical cues required for tissue morphogenesis, differentiation, adipogenesis and homeostasis (Frantz *et al.*, 2010; Johnston and Abbott, 2022). Overall, further functional studies of marine mammal blubber considering the ratio of circulating versus endogenous tissue-specific proteins, as well as changes in perfusion and thus connectivity with the rest of the body, are needed to synthesize *in vivo* and *in vitro* data, as this tissue can provide important insights into evolution, health and conservation of marine mammals.

### Towards health assessments in baleen whales?

These proteomic data suggest that blubber biopsy sampling of superficial, less invasive blubber layers, with subsequent short-term storage under field conditions, followed by long-term storage at −20°C can provide previously unstudied opportunities for protein biomarker assessments. The remotely obtained biopsy samples from the minke whales were stored on ice immediately after collection, but a small amount of tissue degradation is possible because of the delay between sample collection and sample freezing. Ideally, samples should be collected and immediately frozen (Van Cise *et al.*, 2024). However, the logistics of remote field sampling prohibit this in many cases, so the wide range of proteins and peptides identified here across a variety of metabolic pathways and processes suggests that proteomics is a robust tool for comparative studies to investigate overall tissue function using this biopsy sampling approach. Importantly, for quantitative purposes, such samples should only be compared to others collected and stored under similar conditions to avoid misinterpreting variation between individuals as biologically relevant when sampling artefacts could account for some differences. As this approach can also provide valuable information on physiological processes from post-mortem samples (Kershaw *et al.*, 2018), understanding the impacts of sample storage on proteomic profiles as well as the impacts of autolysis on specific protein groups in the tissue will be important moving forward for the wide-scale application of these techniques.

The over-representation analysis carried out here is used to identify if a set of variables such as a protein or gene set, or metabolic pathway, is more prevalent than expected by chance. This is a powerful approach when using biologically complex samples, but when interpreting the output, it is important to consider that the analysis can be biased when using a non-target species as the background for comparison. More marine mammal tissue-specific information is therefore required to overcome the current limitations of these ‘omics applications. There were >400 different proteins identified in these extracts, and given the rapid evolution of proteomics in terms of both sample preparation protocols and technologies that allow for increased detection and measurement accuracy, we can expect modern proteomics to play a vital role in molecular mechanism detection (Cui *et al.*, 2022). Untargeted proteomic approaches will prove to be especially powerful for identifying important tissue processes involved in whole-body maintenance in cetaceans. Moving forward, the targeted quantification of different hormones and proteins involved in various specific metabolic pathways within blubber tissue could lead to the further development of potential new protein markers of immune system function, oxidative stress and lipid metabolism of interest. Explaining and quantifying the natural variability in both targeted and non-targeted approaches in the context of different life history strategies or periods of the life cycle is the next step in cetacean proteomics research, and specifically its application to population health assessments of baleen whales.

## Supplementary Material

Web_Material_coae059

## Data Availability

All proteomic data are available in the supplementary materials.
