# Supplementary material for: Proteome profiling reveals opportunities to investigate biomarkers of oxidative stress and immune responses in blubber biopsies from free-ranging baleen whales
Source: Conserv Physiol. 2024 Aug 19;12(1):coae059. doi: 10.1093/conphys/coae059 (PMC11332026; doi:10.1093/conphys/coae059)
Supplement: Web_Material_coae059 [file web_material_coae059.pdf]

Supplementary Materials

Proteome profiling reveals opportunities to investigate biomarkers of oxidative stress and immune responses in blubber biopsies from free ranging baleen whales

Joanna Kershaw[1,2,3*], Christian. Ramp[1,3], Richard. Sears[3], Ailsa. Hall[1], Davina Derous[2]

[1.] Sea Mammal Research Unit, University of St Andrews, St Andrews, Scotland.

[2.] School of Biological Sciences, University of Aberdeen, Aberdeen, Scotland.

[3.] Mingan Island Cetacean Study, Saint Lambert, Quebec, Canada

*Corresponding author

Table S1. Significantly enriched Gene Ontology (GO): Biological Processes in the blubber extracts. Biological processes were automatically allocated into functionally groups by ClueGO. A biological process can be a member of more than one, larger group and will thus appear multiple times in the table below.

GO term	Adjusted p-value	GO group	Gene of associated proteins found
cellular aldehyde metabolic process	0.001	Group00	AKR1A1, ALDH1A1, ALDH2, ALDH9A1, GLO1, HYI, IDH1, PARK7, TALDO1, TKT, TPI1
muscle structure development	0.010	Group01	ACTA1, ACTB, ACTG1, ACTN4, CALR, CD81, CD9, CFH, COL6A3, CRYAB, CSRP1, EHD1, EHD2, FKBP1A, FLNB, GPX1, HNRNPU, ITGA7, ITGB1, LAMA2, LAMA5, LAMB1, LAMB2, LAMC1, LGALS1, LMNA, MYH9, MYL6, NID1, OBSL1, PTBP1, RHOA, SOD2, SRI, TAGLN, UCHL1
glutathione metabolic process	0.042	Group02	GLO1, GPX1, GSS, GSTA4, IDH1, PARK7, SOD1, SOD2
cell recognition	0.029	Group03	ALDOA, C4BPA, CCT3, CCT5, CCT7, CD36, CD81, CD9, CSRP1, FETUB, MSN, PTX3, RPSA, TCP1

tissue development	0.007	Group04	ACAT1, ACTA1, ACTA2, ACTB, ACTG1, ADIPOQ, AGT, ANXA1, BGN, C3, CA2, CALR, CBR1, CD151, CDC42, CLEC3B, CNRIP1, COL12A1, COL18A1, COL1A1, COL1A2, COL6A1, CSRP1, CTSZ, FABP5, FASN, FERMT2, FKBP1A, FLNB, GLIPR2, GPI, GPX1, HNRNPU, IFNG, IQGAP1, ITGA7, ITGB1, KRT1, KRT14, KRT2, KRT5, KRT75, KRT9, LAMA4, LAMA5, LAMB1, LAMB2, LAMC1, LMNA, LPL, MMP2, MSN, MYL6, MYO1E, OBSL1, OGN, PFN1, PGK1, POSTN, RAB10, RAB1A, RAB1B, RAC1, RAP1A, RAP1B, RHOA, S100A4, SCIN, SERPINH1, SFN, SOD1, TAGLN, TAGLN2, TGFBI, TNC, TNXB, TPT1, TUBB, UBB, VCL, VIM
inflammatory response to antigenic stimulus	0.013	Group05	A2M, C3, CD81, GPX1, IGHA2, IGHG1, KARS1, PARK7, PSMA1, PSMB4
protein folding in endoplasmic reticulum	0.000	Group06	CALR, CANX, HSP90B1, HSPA5, P4HB, PDIA3
regulation of programmed cell death	0.025	Group07	ACTB, ACTN4, ADIPOQ, AGT, AKR1A1, ALB, ALKBH1, ANXA1, ARF4, ARHGDIA, ARL6IP1, CALR, CAMK2D, CFL1, CLU, CRYAB, CTSD, DDAH2, EIF5A, ENO1, ERP29, ETFA, FASN, GBE1, GLO1, GPI, GPX1, GSN, HSP90AA1, HSP90AB1, HSP90B1, HSPA1A, HSPA1B, HSPA5, HSPA9, HSPB1, HSPD1, IFNG, ITGB1, KNG1, LGALS1, LMNA, MIF, MMP2, P4HB, PARK7, PDIA3, PIP, PPIA, PRDX2, PRDX3, PTPA, RHOA, RPL11, RPS3, SCIN, SFN, SOD1, SOD2, TMEM109, TPT1, UBB, VCP, YWHAZ
maintenance of location	0.020	Group08	ABHD5, ALB, APOA1, APOB, C3, CALR, CAMK2D, CD36, CLU, CSRP1, EHD1, FKBP1A, FTL, GSN, HNRNPU, HSP90B1, HSPA5, LPL, PARK7, SCIN, SRI, THY1, YWHAB
detection of stimulus	0.024	Group09	PIP, RPSA, SOD2

regulation of biological quality	0.000	Group10	ACTA2, ACTB, ACTG1, ADIPOQ, AFM, AGT, ALDH1A1, ALDH9A1, ALDOA, ANXA1, ANXA2, APOA1, APOA2, ARF4, ATP1A1, ATP5F1B, ATP5IF1, C2CD2L, CA2, CACNA2D1, CALR, CAMK2D, CCT3, CCT5, CCT7, CD36, CD59, CD81, CD9, CDC42, CFH, CFL1, CIRBP, CLU, COL1A2, COTL1, CPB2, CRYAB, CSRP1, CTSG, CTSZ, DCN, DLD, DTD2, F12, F13A1, FBLN1, FERMT2, FLNB, GAPDH, GDI1, GLUD1, GPI, GPX1, GSN, HADHA, HBB, HCRT, HNRNPC, HNRNPR, HNRNPU, HSP90AA1, HSP90AB1, HSPA1A, HSPA1B, HSPA8, HSPB1, HSPD1, IFNG, ITGA7, ITGB1, KIF5B, KNG1, KRT1, LAMP2, LMNA, MAOA, MMP2, MSN, MYH9, P4HB, PARK7, PDIA3, PFN1, PFN2, PLG, POSTN, PPIA, PPIB, PRDX2, PRDX3, PTGES3, RAB11A, RAB1A, RAB39A, RAB7A, RAC1, RAP1A, RAP1B, RHOA, RPL11, RPS5, SCIN, SERPINA1, SERPINA7, SERPINC1, SERPIND1, SOD1, SOD2, SPTAN1, SPTB, SRI, SYNCRIP, TAPBP, TCP1, TF, THRAP3, TLN1, TUBB, UBB, VCL, VCP, VIM, VPS35, VWF, YWHAG, YWHAZ
reactive nitrogen species metabolic process	0.003	Group11	CD36, CLU, CYB5R3, DDAH2, HBB, HSP90AA1, HSP90AB1, IFNG, PTX3, RAC1, SOD2
tricarboxylic acid cycle	0.001	Group12	ACO2, CFH, IDH1, MDH1, MDH2, SDHA, SDHB, SUCLA2
actin filament-based process	0.000	Group13	ACTA1, ACTA2, ACTB, ACTG1, ACTN4, ALDOA, APOA1, ARPC4, ATP1A1, CACNA2D1, CALR, CAMK2D, CAP1, CDC42, CFL1, COTL1, CSRP1, DPYSL3, EHD2, FERMT2, FLNB, FSCN1, GSN, HSP90B1, IQGAP1, ITGB1, MYH9, MYL6, MYO1C, MYO1E, OBSL1, PFN1, PFN2, RAC1, RAN, RHOA, SCIN, SORBS1, SPTAN1, SPTB, SRI, TAGLN2, TF, TLN1, TNXB, TPM4
actin cytoskeleton organization	0.001	Group13	ACTA1, ACTB, ACTG1, ACTN4, ALDOA, APOA1, ARPC4, CALR, CAP1, CDC42, CFL1, COTL1, CSRP1, DPYSL3, EHD2, FERMT2, FLNB, FSCN1, GSN, HSP90B1, IQGAP1, ITGB1, MYH9, MYO1C, MYO1E, OBSL1, PFN1, PFN2, RAC1, RAN,

			RHOA, SCIN, SORBS1, SPTAN1, SPTB, TAGLN2, TF, TLN1, TNXB, TPM4
biological process involved in symbiotic interaction	0.006	Group14	CD81, CDC42, CFL1, CTSG, EEF1A1, FASN, GAPDH, GPX1, GSN, HSP90AB1, HSPA1A, HSPA1B, HSPA8, HSPD1, ITGB1, LGALS1, P4HB, PLG, PPIA, PPIB, PTX3, RAB7A, RPSA
biological process involved in interaction with symbiont	0.041	Group14	CDC42, CFL1, CTSG, EEF1A1, FASN, GAPDH, GPX1, HSPA8, HSPD1, PLG, PPIB, PTX3
response to lipoprotein particle	0.047	Group15	CD36, CD81, CD9, CDH13, ITGB1, LPL
lipoprotein particle mediated signaling	0.027	Group15	CD36, CDH13, LPL
response to stimulus	0.000	Group16	A2M, ACAT1, ACO2, ACSL1, ACTA1, ACTA2, ACTB, ACTG1, ACTN4, ADIPOQ, AGT, AKR1A1, ALB, ALDH1A1, ALKBH1, ANXA1, ANXA2, AOC3, APOA1, APOA2, APOA4, APOB, APOD, ARHGDIA, ARHGDIB, ATP1A1, ATP5F1A, ATP5F1B, ATP5IF1, BRAP, C2CD2L, C3, C4A, C4BPA, C5, C6, C7, C8A, C9, CA2, CABP1, CACNA2D1, CALR, CAMK2D, CANX, CAP1, CBR1, CCT5, CD151, CD163, CD36, CD59, CD81, CD9, CDC42, CDH13, CFB, CFH, CFI, CFL1, CIRBP, CLEC3B, CLU, CNRIP1, COL15A1, COL18A1, COL1A1, COL1A2, COL6A1, COL6A3, COTL1, CPB2, CRYAB, CSRP1, CTSD, CTSG, DCN, DDAH2, DPYSL2, DPYSL3, EEF1A1, EHD1, EIF5A, ENO1, ERP29, ETFA, F12, F13A1, FABP4, FABP5, FASN, FBLN1, FBLN5, FERMT2, FKBP10, FKBP1A, FLNB, FMOD, FSCN1, GAPDH, GBP6, GDI1, GLIPR2, GLUL, GNB2, GOT1, GPD1, GPI, GPX1, GPX3, GSN, GSS, GSTA4, H2BC21, HADHA,

			HADHB, HBA1, HBB, HBE1, HCRT, HMGN1, HNRNPC, HNRNPU, HP, HPX, HSP90AA1, HSP90AB1, HSP90B1, HSPA1A, HSPA1B, HSPA5, HSPA6, HSPA8, HSPB1, HSPD1, HSPG2, IDH1, IFNG, IGFALS, IGHA1, IGHA2, IGHG1, IGHG3, IGHG4, IGHM, IGHV3-23, IGHV4OR15-8, IGKC, IGLC1, IGLV1-44, IGLV1-51, IGLV2-23, IGLV3-1, IQGAP1, ITGA7, ITGB1, ITIH4, KARS1, KCTD12, KIF5B, KNG1, KRT1, KRT5, LAMA2, LAMA5, LAMB1, LAMB2, LAMC1, LAMP2, LANCL1, LGALS1, LMNA, LPL, LYZ, MAOA, MCAM, MELTF, MFAP4, MGLL, MIF, MMP2, MSN, MYH9, MYO1C, MYO1E, NCF2, NCL, NID1, NPEPPS, ODC1, OGN, OLFML1, OLFML3, P4HB, PARK7, PDIA3, PDIA4, PEBP1, PGK1, PIP, PKM, PLG, PLIN1, POSTN, PPIA, PPIB, PRDX1, PRDX2, PRDX3, PRDX4, PRDX6, PRELP, PRXL2A, PSMA1, PSMA2, PSMA6, PSMA7, PSMB2, PSMB4, PSMB5, PTBP1, PTGES3, PTX3, PYROXD1, RAB10, RAB14, RAB1A, RAB39A, RAB7A, RAC1, RAN, RAP1A, RAP1B, RASSF5, RHOA, RPL11, RPS3, RPSA, RSU1, S100A11, S100A4, SERPINA1, SERPINC1, SERPIND1, SERPINF1, SERPINH1, SFN, SLC27A1, SLPI, SOD1, SOD2, SORBS1, SRI, SYNCRIP, TAPBP, TF, TGFBI, THBS4, THY1, TIMP2, TLN1, TMEM109, TMX1, TNC, TNXB, TPT1, TRIB2, TUBA1B, TUBB, TUFM, UBA1, UBB, UBE2K, UCHL1, UFC1, VCL, VCP, VIM, VPS35, VWF, YWHAB, YWHAG, YWHAZ
response to stress	0.000	Group16	A2M, ACAT1, ACO2, ACTB, ACTG1, ADIPOQ, AGT, ALB, ALKBH1, ANXA1, ANXA2, AOC3, APOA1, APOA4, APOD, ATP5IF1, C3, C4A, C4BPA, C5, C6, C7, C8A, C9, CALR, CANX, CD151, CD163, CD36, CD59, CD81, CD9, CDC42, CFB, CFH, CFI, CIRBP, CLU, CNRIP1, COL18A1, COL1A1, COTL1, CPB2, CRYAB, CSRP1, CTSG, DPYSL3, ENO1, ERP29, F12, F13A1, FABP4, FASN, FBLN1, FBLN5, FERMT2, FKBP10, FLNB, FSCN1, GAPDH, GBP6, GLUL, GPI, GPX1, GPX3, GSN, GSS, H2BC21, HBA1, HBB, HCRT, HMGN1, HNRNPC, HP, HPX, HSP90AA1, HSP90AB1, HSP90B1, HSPA1A, HSPA1B, HSPA5, HSPA6, HSPA8, HSPB1, HSPD1, HSPG2, IDH1, IFNG,

			IGHA1, IGHA2, IGHG1, IGHG3, IGHG4, IGHM, ITGB1, ITIH4, KARS1, KIF5B, KNG1, KRT1, LAMB2, LAMC1, LAMP2, LGALS1, LMNA, LPL, LYZ, MCAM, MELTF, MGLL, MIF, MMP2, MYH9, MYO1C, MYO1E, NCF2, NPEPPS, P4HB, PARK7, PDIA3, PDIA4, PGK1, PLG, PLIN1, POSTN, PPIA, PRDX1, PRDX2, PRDX3, PRDX4, PRDX6, PRELP, PSMA1, PSMA6, PSMA7, PSMB4, PSMB5, PTX3, PYROXD1, RAB14, RAB1A, RAC1, RHOA, RPS3, RPSA, SERPINA1, SERPINC1, SERPIND1, SERPINH1, SFN, SLC27A1, SLPI, SOD1, SOD2, SYNCRIP, TF, THBS4, THY1, TLN1, TMEM109, TMX1, TNC, TNXB, TPT1, TUBB, UBA1, UBE2K, UCHL1, UFC1, VCL, VCP, VIM, VPS35, VWF, YWHAZ
response to chemical	0.000	Group16	A2M, ACAT1, ACSL1, ACTA1, ACTB, ACTG1, ACTN4, ADIPOQ, AKR1A1, ALB, ALDH1A1, ALKBH1, ANXA1, AOC3, APOA1, APOA2, APOA4, APOD, ATP1A1, ATP5F1A, ATP5F1B, C2CD2L, C5, CA2, CACNA2D1, CALR, CAMK2D, CANX, CBR1, CD36, CD81, CD9, CDC42, CDH13, CLEC3B, CLU, CNRIP1, COL18A1, COL1A1, COL1A2, COL6A1, COL6A3, CPB2, CRYAB, CTSD, CTSG, DPYSL3, EIF5A, ENO1, F12, FABP4, FASN, FBLN5, FLNB, GAPDH, GBP6, GDI1, GLUL, GOT1, GPD1, GPI, GPX1, GPX3, GSN, GSS, GSTA4, HADHA, HADHB, HBA1, HBB, HBE1, HNRNPC, HNRNPU, HP, HPX, HSP90AA1, HSP90AB1, HSP90B1, HSPA1A, HSPA1B, HSPA5, HSPA6, HSPA8, HSPB1, HSPD1, HSPG2, IDH1, IFNG, IGHA2, IQGAP1, ITGB1, ITIH4, KIF5B, LAMA2, LAMB2, LAMC1, LANCL1, LMNA, LPL, MELTF, MIF, MMP2, MSN, MYO1C, MYO1E, NCL, NPEPPS, P4HB, PARK7, PDIA3, PGK1, PIP, PKM, POSTN, PPIA, PPIB, PRDX1, PRDX2, PRDX3, PRDX4, PRDX6, PRXL2A, PSMB2, PTGES3, PYROXD1, RAB10, RAC1, RAN, RAP1A, RAP1B, RHOA, RPS3, RPSA, S100A4, SERPIND1, SERPINF1, SERPINH1, SLC27A1, SLPI, SOD1, SOD2, SORBS1, SRI, SYNCRIP, TF, THBS4, TIMP2, TNC, TUBA1B, TUFM, UBE2K, UCHL1, VCP, VIM, VPS35, YWHAG

catabolic process	0.000	Group17	ABHD5, ACAT1, ACSL1, ADIPOQ, AKR1A1, ALDH1A1, ALDH2, ALDOA, ANXA2, APOA2, APOA4, APOB, ATP5IF1, BLVRB, C4BPA, CALR, CANX, CAPNS1, CD36, CD81, CIRBP, CLU, CPB2, CRMP1, CTSD, CTSZ, DCN, DDAH2, DLD, DPYSL2, DPYSL3, ECHS1, EEF1A1, ENO1, ETFA, FLNB, FUT2, GAPDH, GLUD1, GLUL, GOT1, GPD1, GPD2, GPI, GPX1, GPX3, HADHA, HADHB, HBA1, HBB, HBE1, HINT2, HNRNPC, HNRNPR, HNRNPU, HP, HSP90AA1, HSP90AB1, HSP90B1, HSPA1A, HSPA1B, HSPA5, HSPA8, HSPB1, IDH1, IFNG, ILVBL, ITGB1, LAMP2, LDHA, LPL, MAOA, MGLL, MMP2, MRC2, MSN, NPEPPS, ODC1, PAFAH1B2, PARK7, PGAM1, PGK1, PGK2, PKM, PLIN1, PRDX1, PRDX2, PRDX3, PRDX4, PRDX6, PSMA1, PSMA2, PSMA3, PSMA4, PSMA5, PSMA6, PSMA7, PSMB1, PSMB2, PSMB3, PSMB4, PSMB5, PSMB6, PSMB9, PSME1, RAB1A, RAB1B, RAB39A, RAB7A, RPL11, SH3BGRL, SND1, SUCLA2, SYNCRIP, THRAP3, TIMP2, TPI1, TRIB2, TRNT1, UBA1, UBB, UBE2K, UCHL1, UFC1, VCP, VIM, VPS35
cellular catabolic process	0.000	Group17	ABHD5, ACAT1, ACSL1, ADIPOQ, AKR1A1, ALDH2, ANXA2, APOA2, APOA4, APOB, ATP5IF1, BLVRB, CALR, CAPNS1, CD36, CIRBP, CLU, CRMP1, CTSD, DCN, DDAH2, DLD, DPYSL2, DPYSL3, ECHS1, EEF1A1, ETFA, GAPDH, GLUD1, GLUL, GOT1, GPD1, GPX1, GPX3, HADHA, HADHB, HBA1, HBB, HBE1, HNRNPC, HNRNPR, HNRNPU, HP, HSP90AA1, HSPA1A, HSPA1B, HSPA8, HSPB1, IDH1, IFNG, ILVBL, LAMP2, LPL, MAOA, MGLL, NPEPPS, PAFAH1B2, PARK7, PRDX1, PRDX2, PRDX3, PRDX4, PRDX6, RAB1A, RAB1B, RAB39A, RAB7A, SND1, SUCLA2, SYNCRIP, THRAP3, TIMP2, TRNT1, UCHL1, UFC1, VCP, VIM, VPS35
organic substance catabolic process	0.000	Group17	ABHD5, ACAT1, ADIPOQ, AKR1A1, ALDH1A1, ALDH2, ALDOA, ANXA2, APOA2, APOA4, APOB, ATP5IF1, BLVRB, C4BPA, CALR, CANX, CD81, CIRBP, CLU, CPB2, CRMP1, CTSD, CTSZ, DDAH2, DLD, DPYSL2, DPYSL3, ECHS1, EEF1A1, ENO1, ETFA, FLNB, FUT2, GAPDH, GLUD1, GLUL,

			GOT1, GPD1, GPD2, GPI, GPX1, GPX3, HADHA, HADHB, HBA1, HBB, HBE1, HINT2, HNRNPC, HNRNPR, HNRNPU, HP, HSP90AA1, HSP90AB1, HSP90B1, HSPA1A, HSPA1B, HSPA5, HSPA8, IDH1, IFNG, ILVBL, LAMP2, LDHA, LPL, MAOA, MGLL, MMP2, MSN, NPEPPS, ODC1, PAFAH1B2, PARK7, PGAM1, PGK1, PGK2, PKM, PLIN1, PRDX1, PRDX2, PRDX3, PRDX4, PRDX6, PSMA1, PSMA2, PSMA3, PSMA4, PSMA5, PSMA6, PSMA7, PSMB1, PSMB2, PSMB3, PSMB4, PSMB5, PSMB6, PSMB9, PSME1, RAB7A, RPL11, SH3BGRL, SND1, SUCLA2, SYNCRIP, THRAP3, TIMP2, TPI1, TRIB2, TRNT1, UBA1, UBB, UBE2K, UCHL1, VCP, VIM, VPS35
regulation of plasma lipoprotein particle levels	0.010	Group18	ADIPOQ, AGT, ANXA2, APOA1, APOA2, APOA4, APOB, CD36, EHD1, LPL
plasma lipoprotein particle clearance	0.032	Group18	ADIPOQ, ANXA2, APOA1, APOA2, APOB, CD36, EHD1
regulation of lipid localization	0.003	Group18	ABHD5, ACSL1, ADIPOQ, AGT, ANXA2, APOA1, APOA2, APOA4, APOB, C3, CD36, CSRP1, EHD1, LPL, MIF, SAR1B
positive regulation of biological process	0.000	Group19	A2M, ABHD5, ACSL1, ACTA1, ACTA2, ACTB, ACTG1, ACTN4, ADIPOQ, AGT, ANXA1, ANXA2, APOA1, APOA2, APOA4, APOB, ARF4, ARL6IP1, ATP1A1, ATP5F1A, ATP5F1B, ATP5IF1, C2CD2L, C3, C4A, C4BPA, C5, C6, C7, C8A, C9, CA2, CACNA2D1, CALR, CAMK2D, CAND1, CAPNS1, CAVIN1, CBR1, CCT3, CCT5, CCT7, CD151, CD36, CD59, CD81, CDC42, CDH13, CFB, CFH, CFI, CFL1, CIRBP, CLEC3B, CLU, COL1A1, CPB2, CSRP1, CTSD, CTSG, DCN, DDAH2, DPYSL3, EEF2, EHD1, EHD2, EIF5A, ENO1, ERP29, ETFA, F12, FABP4, FABP5, FASN, FERMT2, FKBP1A, FLNB, FSCN1, GAPDH, GC, GDI1, GLIPR2, GLUD1, GPD1, GPI, GPX1, GSN, HADHA, HBB, HCRT, HMGN1, HNRNPA2B1, HNRNPC, HNRNPR, HNRNPU, HPX, HSP90AA1, HSP90AB1,

			HSPA1A, HSPA1B, HSPA5, HSPA8, HSPB1, HSPD1, HSPG2, IFNG, IGHA1, IGHA2, IGHG1, IGHG3, IGHG4, IGHM, IGKC, IGLC1, ILF2, IQGAP1, ITGB1, KARS1, KIF5B, KNG1, KRT1, KRT2, LAMA2, LAMB1, LAMB2, LAMC1, LGALS1, LMNA, LPL, LUM, MAOA, MCAM, MELTF, MIF, MMP2, MSN, MYH9, MYO1C, MYO1E, NCL, NID1, NIPSNAP2, NPEPPS, OBSL1, ODC1, P4HB, PAFAH1B2, PARK7, PDIA3, PFN1, PFN2, PIP, PKM, PLG, POSTN, PPIA, PPIB, PRDX2, PRDX3, PRDX6, PSMA7, PSME1, PTBP1, PTGES3, PTPA, PTX3, RAB11A, RAB1A, RAB1B, RAB3D, RAB7A, RAC1, RAN, RAP1A, RAP1B, RASSF5, RHOA, RPL11, RPS2, RPS3, RPSA, RSU1, S100A11, S100A4, SAR1B, SCIN, SERPINF1, SFN, SH3BGRL, SLC27A1, SOD1, SOD2, SORBS1, SPON1, SRI, SYNCRIP, TCP1, TF, THBS4, THRAP3, THY1, TNC, TNXB, TRIB2, UBB, UBE2K, VCP, VIM, VPS35, VWF
regulation of response to stimulus	0.024	Group19	A2M, ACTA2, ACTB, ACTG1, ACTN4, ADIPOQ, AGT, ANXA1, ANXA2, APOA1, APOD, ARHGDIA, ARHGDIB, BRAP, C3, C4A, C4BPA, C5, C6, C7, C8A, C9, CALR, CAMK2D, CD36, CD59, CD81, CD9, CDC42, CDH13, CFB, CFH, CFI, CLU, CNRIP1, COL1A1, CPB2, CTSD, CTSG, DCN, EIF5A, ENO1, ERP29, ETFA, F12, FABP4, FABP5, FASN, FBLN1, FBLN5, FERMT2, FKBP1A, FLNB, GAPDH, GLIPR2, GPI, GPX1, HNRNPC, HPX, HSP90AA1, HSP90AB1, HSPA1A, HSPA1B, HSPA5, HSPB1, HSPD1, IFNG, IGHA1, IGHA2, IGHG1, IGHG3, IGHG4, IGHM, IGKC, IGLC1, IQGAP1, ITGB1, KARS1, KCTD12, KNG1, KRT1, LAMA2, LAMB1, LAMB2, LAMC1, LGALS1, LMNA, LPL, MAOA, MGLL, MIF, MYO1C, MYO1E, NCL, NID1, P4HB, PARK7, PDIA3, PEBP1, PLG, POSTN, PPIA, PRDX1, PRDX2, PSMA1, PSMA6, PSMA7, PSMB4, PTBP1, RAB7A, RAC1, RAP1A, RAP1B, RHOA, RPL11, RPS3, RPSA, S100A4, SERPINC1, SOD1, SOD2, SORBS1, THBS4, THY1, TNC, TNXB, TPT1, UBB, UBE2K, UCHL1, VCP, VPS35, VWF, YWHAB, YWHAG, YWHAZ

positive regulation of metabolic process	0.040	Group19	ABHD5, ACSL1, ACTA1, ACTA2, ACTB, ACTG1, ACTN4, ADIPOQ, AGT, ANXA1, ANXA2, APOA1, APOA2, APOA4, APOB, ARF4, ATP5IF1, C3, C4BPA, C5, CALR, CAND1, CBR1, CCT3, CCT5, CCT7, CD36, CD81, CDC42, CDH13, CFH, CIRBP, CLEC3B, CLU, COL1A1, CSRP1, CTSD, DCN, DDAH2, EEF2, EIF5A, ENO1, ERP29, F12, FABP4, FABP5, FASN, FERMT2, FKBP1A, FLNB, GAPDH, GC, GPD1, GPI, GSN, HADHA, HBB, HCRT, HMGN1, HNRNPA2B1, HNRNPC, HNRNPR, HNRNPU, HPX, HSP90AA1, HSP90AB1, HSPA1A, HSPA1B, HSPA5, HSPA8, HSPB1, HSPD1, IFNG, IGHA1, IGHA2, ILF2, IQGAP1, KARS1, LMNA, LPL, LUM, MELTF, MIF, MSN, MYH9, MYO1C, NCL, PAFAH1B2, PARK7, PFN2, PIP, PKM, POSTN, PPIA, PRDX6, PSME1, PTBP1, PTGES3, PTPA, PTX3, RAB1A, RAB1B, RAB7A, RAC1, RAP1A, RASSF5, RHOA, RPL11, RPS2, RPS3, RPSA, SH3BGRL, SLC27A1, SOD1, SOD2, SORBS1, SPON1, SYNCRIP, TCP1, TF, THBS4, THRAP3, TNC, TNXB, TRIB2, UBB, UBE2K, VCP, VIM, VPS35
positive regulation of response to stimulus	0.000	Group19	A2M, ACTA2, ACTB, ACTG1, ACTN4, ADIPOQ, AGT, ANXA1, APOA1, C3, C4A, C4BPA, C5, C6, C7, C8A, C9, CALR, CD36, CD59, CD81, CDC42, CDH13, CFB, CFH, CFI, CLU, COL1A1, CPB2, CTSD, CTSG, DCN, EIF5A, ERP29, ETFA, F12, FABP4, FABP5, FASN, FERMT2, FKBP1A, FLNB, GAPDH, GLIPR2, GPX1, HNRNPC, HPX, HSP90AA1, HSP90AB1, HSPA1A, HSPA1B, HSPB1, HSPD1, IFNG, IGHA1, IGHA2, IGHG1, IGHG3, IGHG4, IGHM, IGKC, IGLC1, IQGAP1, ITGB1, KARS1, KRT1, LAMA2, LAMB1, LAMB2, LAMC1, LGALS1, LPL, MAOA, MIF, MYO1C, MYO1E, NID1, PARK7, PDIA3, PLG, PPIA, PRDX2, PSMA7, PTBP1, RAC1, RAP1A, RAP1B, RHOA, RPL11, RPS3, RPSA, S100A4, SOD1, SORBS1, THBS4, THY1, TNXB, UBB, UBE2K, VCP, VPS35, VWF
homeostatic process	0.000	Group20	ABHD5, ACSL1, ACTB, ACTG1, ADIPOQ, AGT, ALB, ALDH1A1, ALDOA, ANXA1, APOA1, APOA2, APOA4, APOB, ATP1A1, ATP5F1B, ATP5IF1, C2CD2L, CA2, CALR, CAMK2D,

			CD36, CFH, CLU, CP, CPB2, EHD1, FABP4, FABP5, FASN, FKBP1A, FLNB, FTL, GOT1, GPI, GPX1, HADHA, HCRT, HPX, HSP90B1, HSPA1A, HSPA1B, HSPA9, HSPB1, IFNG, IGHA1, IGHA2, IGHG3, IGKC, ITGB1, KRT1, LAMA2, LAMA4, LAMC1, LAMP2, LPL, LYZ, MELTF, MIF, PARK7, PIP, PRDX1, PRDX2, PRDX3, PRDX4, PRDX6, RAB39A, RAB3D, RAB7A, SAR1B, SERPINF1, SLC27A1, SOD1, SOD2, SRI, TF, THY1, TPT1, UBB, UBE2K, VCL
multicellular organismal-level homeostasis	0.001	Group20	ACSL1, ACTB, ACTG1, ADIPOQ, ALB, ALDH1A1, ANXA1, ATP5IF1, CD36, CFH, CLU, FABP4, FABP5, FASN, GPI, GPX1, HADHA, HCRT, HSPA1A, HSPA1B, HSPA9, HSPB1, IGHA1, IGHA2, IGHG3, IGKC, ITGB1, KRT1, LAMA2, LAMA4, LAMC1, LYZ, MELTF, MIF, PIP, PRDX1, PRDX2, RAB3D, RAB7A, SLC27A1, SOD1, SOD2, TF, UBB, VCL
anatomical structure homeostasis	0.001	Group20	ACTB, ACTG1, ALB, ALDH1A1, CFH, HSPB1, IGHA1, IGHA2, IGHG3, IGKC, ITGB1, KRT1, LAMA2, LAMC1, LYZ, MELTF, PIP, PRDX1, RAB3D, RAB7A, SOD1, TF, VCL
tissue homeostasis	0.001	Group20	ACTB, ACTG1, ALB, ALDH1A1, CFH, HSPB1, IGHA1, IGHA2, IGHG3, IGKC, ITGB1, KRT1, LAMA2, LAMC1, LYZ, MELTF, PIP, PRDX1, RAB3D, RAB7A, SOD1, TF, VCL
regulation of localization	0.000	Group21	ABHD5, ACSL1, ACTB, ACTG1, ACTN4, ADIPOQ, AGT, ANXA1, ANXA2, APOA1, APOA2, APOA4, APOB, APOD, ARHGDIA, ARL6IP1, ATP1A1, ATP5IF1, C2CD2L, C3, C4A, CA2, CABP1, CACNA2D1, CALR, CAMK2D, CCT3, CCT5, CCT7, CD151, CD36, CD81, CDC42, CDH13, CFH, CLU, CPB2, CRYAB, CSRP1, CTSD, EHD1, EHD2, ERP29, FABP5, FERMT2, FKBP1A, FLNB, GDI1, GLUD1, GLUL, GNB2, GSN, HADHA, HCRT, HNRNPC, HNRNPU, HSP90AA1, HSP90AB1, HSPA1A, HSPA8, IFNG, ITGB1, KIF5B, KRT5, LMNA, LPL, MIF, MSN, MYO1C, MYO1E, NIPSNAP2, NPEPPS, PARK7, PFN2, PPIA, PTX3, RAB11A, RAB14, RAB3D, RAB7A, RAC1, RAN, RAP1A, RAP1B, RASSF5, SAR1B, SCIN, SFN, SOD1, SORBS1, SRI, TCP1, TF, THY1, TMEM109, VCL, VCP, VPS35, YWHAZ

cellular component organization	0.048	Group21	ACTA1, ACTB, ACTG1, ACTN4, ADIPOQ, AEBP1, AGT, ALDH9A1, ALDOA, ALKBH1, ANXA1, ANXA2, APOA1, APOA2, APOA4, APOB, APOD, ARF4, ARL6IP1, ARPC4, ATP5IF1, C2CD2L, C3, C4A, C9, CALR, CAMK2D, CAND1, CAP1, CAVIN2, CCT3, CCT5, CCT7, CD151, CD36, CD9, CDC42, CDH13, CFH, CFL1, CIRBP, CLU, COL12A1, COL14A1, COL15A1, COL18A1, COL1A1, COL1A2, COTL1, CPB2, CRMP1, CRYAB, CSRP1, CTSD, CTSG, DCN, DPYSL2, DPYSL3, EHD1, EHD2, EIF5A, ENO1, ESYT1, FASN, FBLN1, FBLN5, FERMT2, FKBP10, FKBP1A, FLNB, FMOD, FSCN1, GAPDH, GDI1, GPX1, GSN, H1-0, H2AB1, H2BC21, HMGN1, HNRNPA2B1, HNRNPC, HNRNPU, HSP90AA1, HSP90AB1, HSP90B1, HSPA1A, HSPA1B, HSPA5, HSPA8, HSPA9, HSPD1, IFNG, INPP5J, IQGAP1, ITGB1, KCTD12, KIF5B, KRT1, KRT14, KRT2, KRT5, KRT75, KRT9, LAMA2, LAMA4, LAMA5, LAMB1, LAMB2, LAMC1, LAMP2, LMNA, LPL, LUM, MELTF, MFAP4, MIF, MMP2, MSN, MYH9, MYO1C, MYO1E, NID1, NID2, NIPSNAP2, NPEPPS, OBSL1, PARK7, PDIA3, PFN1, PFN2, PLG, POSTN, PPIA, PRDX3, PRDX4, PRDX6, PTGES3, PTPA, PTX3, RAB10, RAB11A, RAB14, RAB1A, RAB1B, RAB2A, RAB39A, RAB6A, RAB7A, RAC1, RAN, RAP1A, RAP1B, RHOA, RPL11, RPS3, RPS5, RPSA, SAR1B, SCIN, SEPTIN6, SERPINF1, SERPINH1, SFN, SOD1, SOD2, SORBS1, SPTAN1, SPTB, TAGLN2, TAPBP, TBCA, TCP1, TF, TGFBI, THY1, TLN1, TLN2, TNC, TNXB, TPM4, TUBA1B, TUBA1C, TUBA3C, TUBA4A, TUBAL3, TUBB, TUBB4A, UBB, UBE2K, UCHL1, UFC1, UGDH, VAT1, VCL, VCP, VIM, VPS35, YWHAZ
regulation of transport	0.000	Group21	ACSL1, ACTB, ACTG1, ACTN4, ADIPOQ, AGT, ANXA1, ANXA2, APOA1, APOA2, APOA4, APOD, ARHGDIA, ARL6IP1, ATP1A1, ATP5IF1, C2CD2L, C3, C4A, CA2, CABP1, CACNA2D1, CALR, CAMK2D, CD151, CD36, CD81, CDC42, CDH13, CFH, CLU, CPB2, CRYAB, EHD1, EHD2, ERP29, FABP5, FKBP1A, FLNB, GDI1, GLUD1, GNB2, HADHA, HCRT,

			HNRNPC, HSP90AA1, HSPA1A, HSPA8, IFNG, ITGB1, KIF5B, MIF, MSN, MYO1C, MYO1E, NIPSNAP2, NPEPPS, PARK7, PFN2, PPIA, PTX3, RAB11A, RAB3D, RAB7A, RAC1, RAN, RAP1A, RAP1B, SAR1B, SCIN, SFN, SOD1, SORBS1, SRI, TF, THY1, TMEM109, VPS35
regulation of cellular component organization	0.000	Group21	ACTB, ACTG1, ADIPOQ, AEBP1, AGT, ANXA1, ANXA2, APOA1, APOA2, APOA4, APOD, ARF4, ARL6IP1, ATP5IF1, C3, C4A, CALR, CAMK2D, CAND1, CCT3, CCT5, CCT7, CD151, CD36, CDC42, CDH13, CFH, CFL1, CLU, COTL1, CPB2, CRMP1, CRYAB, DCN, DPYSL2, DPYSL3, EHD1, EHD2, EIF5A, ENO1, FERMT2, FSCN1, GDI1, GPX1, GSN, HNRNPA2B1, HNRNPC, HNRNPU, HSP90AA1, HSPA1A, HSPA1B, HSPA5, HSPA8, IFNG, INPP5J, ITGB1, LAMA2, LAMB1, LAMB2, LAMC1, LMNA, MELTF, MSN, MYH9, MYO1C, MYO1E, NID1, OBSL1, PARK7, PFN1, PFN2, PTX3, RAB1B, RAB7A, RAC1, RAP1A, RAP1B, RHOA, RPS3, SAR1B, SCIN, SERPINF1, SFN, SOD1, SPTAN1, SPTB, TCP1, TF, THY1, TLN1, TNC, TNXB, TUBB, TUBB4A, VAT1, VCL, VCP, VIM, VPS35, YWHAZ
positive regulation of cellular component organization	0.001	Group21	AGT, ANXA1, ANXA2, APOA1, APOA2, ATP5IF1, C3, C4A, CALR, CAND1, CCT3, CCT5, CCT7, CD151, CD36, CDC42, CLU, CPB2, DCN, DPYSL3, EHD1, EHD2, EIF5A, FERMT2, FSCN1, GDI1, GPX1, GSN, HNRNPA2B1, HNRNPC, HSP90AA1, HSPA1A, HSPA1B, IFNG, MELTF, MSN, OBSL1, PARK7, PFN1, PFN2, PTX3, RAC1, RAP1A, RAP1B, RHOA, RPS3, SCIN, SERPINF1, SOD1, TCP1, TF, THY1, TNXB, VCP, VPS35
negative regulation of biological process	0.004	Group22	A2M, ABHD5, ACTB, ACTN4, ADIPOQ, AEBP1, AGT, AKR1A1, ALB, ALDH1A1, ALKBH1, ANXA1, ANXA2, APOA1, APOA2, APOA4, APOD, ARF4, ARHGDIA, ARHGDIB, ARL6IP1, ATP1A1, ATP5F1A, ATP5F1B, ATP5IF1, BRAP, C4BPA, C5, CABP1, CALR, CAMK2D, CD36, CD59, CD9, CDC42, CDH13, CFH, CFL1, CIRBP, CLU, COL18A1, COL1A1, COL6A3, CPB2, CRMP1, CRYAB, CSRP1, CTSG, CTSZ, DCN,

			DDAH2, DPYSL3, ENO1, ERP29, F12, FABP4, FABP5, FASN, FBLN1, FERMT2, FETUB, FKBP1A, FLNB, GAPDH, GBE1, GDI1, GLO1, GPI, GPX1, GSN, H1-0, HADHA, HCRT, HINT2, HNRNPA2B1, HNRNPC, HNRNPR, HNRNPU, HP, HSP90AB1, HSP90B1, HSPA1A, HSPA1B, HSPA5, HSPA8, HSPA9, HSPB1, HSPD1, HSPG2, IFNG, IGHA2, INPP5J, IQGAP1, ITGB1, ITIH1, ITIH2, ITIH3, ITIH4, ITIH5, KNG1, KRT1, LAMA4, LMNA, MELTF, MIF, MMP2, MYH9, MYO1C, NCL, OGN, PARK7, PEBP1, PFN1, PFN2, PGK1, PIP, PLG, PODN, POSTN, PPIA, PRDX2, PRDX3, PRDX4, PSMA1, PSMB4, PTBP1, PTPA, PTX3, RAB7A, RAC1, RAN, RAP1A, RAP1B, RASSF5, RHOA, RPL11, RPS3, RPSA, S100A11, SCIN, SERPINA1, SERPINA7, SERPINB1, SERPINC1, SERPIND1, SERPINF1, SERPINH1, SFN, SLC27A1, SLPI, SND1, SOD1, SOD2, SPON1, SPTAN1, SPTB, SRI, SYNCRIP, TGFBI, THBS4, THRAP3, THY1, TIMP2, TMEM109, TNC, TPT1, TRIB2, TUBB4A, UCHL1, VAT1, VCL, VCP, VIM, VPS35, YWHAB, YWHAG, YWHAZ
regulation of molecular function	0.000	Group22	A2M, ACTB, ACTN4, ADIPOQ, AGT, ANXA1, ANXA2, AOC3, APOA1, APOA2, APOA4, ARF4, ARL6IP1, ATP5IF1, CABP1, CACNA2D1, CAMK2D, CAND1, CAP1, CD36, CDC42, CFH, CLU, CNRIP1, COL6A3, CRMP1, CRYAB, CTSD, DDAH2, EEF1A1, ERP29, FABP4, FASN, FERMT2, FETUB, FKBP1A, GAPDH, GPI, GPX1, GSN, H1-0, HMGN1, HNRNPA2B1, HNRNPR, HNRNPU, HP, HSP90AA1, HSP90AB1, HSP90B1, HSPA1A, HSPA1B, HSPB1, HSPD1, IFNG, IGHA2, IQGAP1, ITGB1, ITIH1, ITIH2, ITIH3, ITIH4, ITIH5, KNG1, MIF, NIPSNAP2, PARK7, PEBP1, PFN1, PFN2, PPIA, PRDX2, PRDX3, PSMA3, PSMA6, PSMB9, PSME1, PTGES3, PTPA, PTX3, RAN, RAP1A, RHOA, RPL11, RPS2, RPS3, RSU1, SERPINA1, SERPINA7, SERPINB1, SERPINC1, SERPIND1, SERPINF1, SERPINH1, SFN, SLC27A1, SLPI, SOD1, SOD2, SPON1, SRI, TCP1, THY1, TIMP2, TNXB, TRIB2, UCHL1, VCP, YWHAB, YWHAG

negative regulation of molecular function	0.000	Group22	A2M, ACTB, ADIPOQ, AGT, ANXA1, AOC3, APOA1, APOA2, ARL6IP1, ATP5IF1, CAMK2D, CAND1, CNRIP1, COL6A3, CRMP1, CRYAB, FABP4, FETUB, FKBP1A, GAPDH, GPI, GPX1, HNRNPR, HNRNPU, HP, HSPB1, IFNG, IGHA2, ITIH1, ITIH2, ITIH3, ITIH4, ITIH5, KNG1, PARK7, PEBP1, PPIA, PRDX2, PRDX3, PTPA, PTX3, RPL11, SERPINA1, SERPINA7, SERPINB1, SERPINC1, SERPIND1, SERPINF1, SERPINH1, SFN, SLPI, SRI, THY1, TIMP2, TRIB2, UCHL1, YWHAG
regulation of catalytic activity	0.000	Group22	A2M, ACTB, ADIPOQ, AGT, ANXA1, AOC3, APOA1, APOA2, APOA4, ARF4, ARL6IP1, ATP5IF1, CAMK2D, CAND1, CAP1, CDC42, CLU, COL6A3, CRYAB, CTSD, DDAH2, EEF1A1, ERP29, FABP4, FASN, FERMT2, FETUB, FKBP1A, GAPDH, GPI, GPX1, GSN, HMGN1, HNRNPA2B1, HNRNPR, HNRNPU, HP, HSP90AA1, HSP90AB1, HSP90B1, HSPA1A, HSPB1, HSPD1, IFNG, IGHA2, IQGAP1, ITGB1, ITIH1, ITIH2, ITIH3, ITIH4, ITIH5, KNG1, MIF, PARK7, PEBP1, PPIA, PRDX3, PSMA3, PSMB9, PSME1, PTGES3, PTPA, PTX3, RAP1A, RHOA, RPL11, RPS2, RPS3, RSU1, SERPINA1, SERPINA7, SERPINB1, SERPINC1, SERPIND1, SERPINF1, SERPINH1, SFN, SLC27A1, SLPI, SOD1, SOD2, TCP1, THY1, TIMP2, TNXB, TRIB2, UCHL1, VCP, YWHAB, YWHAG
organonitrogen compound metabolic process	0.000	Group22	A2M, ABHD5, ACAT1, ACSL1, ACSS2, ACTB, ADIPOQ, AEBP1, AGT, AKR1A1, ALDH1A1, ALDH2, ALDH9A1, ALDOA, ALKBH1, ANXA1, ANXA2, AOC3, APOA1, APOA2, APOA4, APOB, APOD, ARL6IP1, ATP5F1A, ATP5F1B, ATP5IF1, ATP5MF, ATP5PB, ATP5PO, BGN, BLVRB, BRAP, C3, C4BPA, CALR, CAMK2D, CAND1, CANX, CAPNS1, CD36, CD81, CDC42, CFB, CFH, CFI, CIRBP, CLEC3B, CLU, CMPK1, COL6A3, CPB2, CRMP1, CRYAB, CTSD, CTSG, CTSZ, DCN, DDAH2, DLD, DPYSL2, DPYSL3, ECHS1, EEF1A1, EEF2, EIF5A, ENO1, ERP29, ETFA, F12, F13A1, FABP4, FABP5, FASN, FBLN1, FERMT2, FETUB, FKBP10, FKBP1A, FUT2, GAPDH, GFM1, GLUD1, GLUL, GOT1, GPD1, GPI, GPX1, GSN, GSS, HCRT, HINT2, HNRNPC, HNRNPR, HNRNPU, HP,

			HPX, HSP90AA1, HSP90AB1, HSP90B1, HSPA1A, HSPA1B, HSPA5, HSPA8, HSPB1, HSPD1, IDH1, IFNG, IGHA2, ILVBL, INPP5J, IQGAP1, ITIH1, ITIH2, ITIH3, ITIH4, ITIH5, KARS1, KLK11, KNG1, KRT1, KRT2, LAMP2, LANCL1, LAP3, LDHB, MAOA, MDH1, MDH2, MELTF, MIF, MMP2, MSN, MYH9, MYO1C, NCL, NPEPPS, ODC1, P4HB, PARK7, PCOLCE, PEBP1, PFN2, PGAM1, PGK1, PIP, PKM, PLG, PPIA, PPIB, PPIC, PRDX3, PRDX4, PSMA1, PSMA2, PSMA3, PSMA4, PSMA5, PSMA6, PSMA7, PSMB1, PSMB2, PSMB3, PSMB4, PSMB5, PSMB6, PSMB9, PSME1, PTBP1, PTPA, PTX3, RAB1A, RAB1B, RAB3D, RAB6A, RAB7A, RAC1, RAN, RAP1A, RASSF5, RHOA, RPL11, RPN1, RPN2, RPS10, RPS2, RPS25, RPS3, RPS5, RPS8, RPSA, SDHA, SDHB, SERPINA1, SERPINA7, SERPINB1, SERPINC1, SERPIND1, SERPINF1, SERPINH1, SFN, SH3BGRL, SLC27A1, SLPI, SOD1, SPON1, SUCLA2, SYNCRIP, TALDO1, TAPBP, THBS4, THY1, TIMP2, TKT, TNXB, TRIB2, TUFM, UBA1, UBB, UBE2K, UCHL1, UFC1, UGDH, VCP, VIM, VPS35, YWHAB, YWHAG, YWHAZ
protein metabolic process	0.000	Group22	A2M, ABHD5, ACTB, ADIPOQ, AEBP1, AGT, ALKBH1, ANXA1, ANXA2, APOA1, APOA2, APOA4, APOB, APOD, ARL6IP1, ATP5IF1, BGN, BRAP, C3, C4BPA, CALR, CAMK2D, CAND1, CANX, CAPNS1, CD36, CD81, CDC42, CFB, CFH, CFI, CIRBP, CLEC3B, CLU, COL6A3, CPB2, CRYAB, CTSD, CTSG, CTSZ, DCN, DLD, EEF1A1, EEF2, EIF5A, ENO1, ERP29, F12, F13A1, FABP4, FASN, FBLN1, FERMT2, FETUB, FKBP10, FKBP1A, FUT2, GAPDH, GFM1, GLUL, GPI, GPX1, GSN, HINT2, HNRNPC, HNRNPR, HNRNPU, HP, HPX, HSP90AA1, HSP90AB1, HSP90B1, HSPA1A, HSPA1B, HSPA5, HSPA8, HSPB1, HSPD1, IFNG, IGHA2, INPP5J, IQGAP1, ITIH1, ITIH2, ITIH3, ITIH4, ITIH5, KARS1, KLK11, KNG1, KRT1, KRT2, LAMP2, LAP3, MELTF, MIF, MMP2, MSN, MYH9, MYO1C, NCL, NPEPPS, ODC1, P4HB, PARK7, PCOLCE, PEBP1, PFN2, PGK1, PIP, PKM, PLG, PPIA, PPIB, PPIC, PRDX3, PRDX4, PSMA1, PSMA2, PSMA3, PSMA4,

			PSMA5, PSMA6, PSMA7, PSMB1, PSMB2, PSMB3, PSMB4, PSMB5, PSMB6, PSMB9, PSME1, PTBP1, PTPA, PTX3, RAB1A, RAB1B, RAB3D, RAB6A, RAB7A, RAC1, RAP1A, RASSF5, RHOA, RPL11, RPN1, RPN2, RPS10, RPS2, RPS25, RPS3, RPS5, RPS8, RPSA, SERPINA1, SERPINA7, SERPINB1, SERPINC1, SERPIND1, SERPINF1, SERPINH1, SFN, SH3BGRL, SLC27A1, SLPI, SOD1, SPON1, SYNCRIP, THBS4, THY1, TIMP2, TNXB, TRIB2, TUFM, UBA1, UBB, UBE2K, UCHL1, UFC1, UGDH, VCP, VIM, VPS35, YWHAB, YWHAG, YWHAZ
protein folding	0.000	Group23	CALR, CANX, CCT3, CCT5, CCT7, CLU, CRYAB, ERP29, FKBP10, FKBP1A, FKBP9, HSP90AA1, HSP90AB1, HSP90B1, HSPA1A, HSPA1B, HSPA5, HSPA6, HSPA8, HSPA9, HSPB1, HSPD1, P4HB, PDIA3, PDIA4, PPIA, PPIB, PPIC, PRDX4, PTGES3, TBCA, TCP1
'de novo' protein folding	0.000	Group23	FKBP1A, HSPA1A, HSPA1B, HSPA5, HSPA6, HSPA8, HSPA9, HSPD1, PTGES3
regulation of protein stability	0.000	Group23	AFM, APOA1, APOA2, CALR, CCT3, CCT5, CCT7, CD81, CLU, CRYAB, GAPDH, GPI, GSN, HSP90AA1, HSP90AB1, HSPA1A, HSPA1B, HSPA8, HSPD1, LAMP2, LMNA, PARK7, PFN1, PFN2, PPIB, PTGES3, RPL11, TCP1, TF, VPS35
protein refolding	0.000	Group23	CRYAB, FKBP1A, HSP90AA1, HSPA1A, HSPA1B, HSPA5, HSPA6, HSPA8, HSPA9, HSPB1, HSPD1
protein folding chaperone	0.000	Group23	CALR, CCT3, CCT5, CCT7, HSP90AA1, HSP90AB1, HSP90B1, HSPA1A, HSPA1B, HSPA5, HSPA6, HSPA8, HSPA9, HSPB1, HSPD1, TCP1
chaperone-mediated protein folding	0.000	Group23	CCT3, CCT5, CCT7, CLU, HSPA1A, HSPA1B, HSPA5, HSPA6, HSPA8, HSPA9, HSPB1, PDIA4, PPIB, PTGES3, TCP1
response to stimulus	0.000	Group24	A2M, ACAT1, ACO2, ACSL1, ACTA1, ACTA2, ACTB, ACTG1, ACTN4, ADIPOQ, AGT, AKR1A1, ALB, ALDH1A1, ALKBH1, ANXA1, ANXA2, AOC3, APOA1, APOA2, APOA4, APOB, APOD, ARHGDIA, ARHGDIB, ATP1A1, ATP5F1A, ATP5F1B, ATP5IF1, BRAP, C2CD2L, C3, C4A, C4BPA, C5, C6, C7, C8A,

			C9, CA2, CABP1, CACNA2D1, CALR, CAMK2D, CANX, CAP1, CBR1, CCT5, CD151, CD163, CD36, CD59, CD81, CD9, CDC42, CDH13, CFB, CFH, CFI, CFL1, CIRBP, CLEC3B, CLU, CNRIP1, COL15A1, COL18A1, COL1A1, COL1A2, COL6A1, COL6A3, COTL1, CPB2, CRYAB, CSRP1, CTSD, CTSG, DCN, DDAH2, DPYSL2, DPYSL3, EEF1A1, EHD1, EIF5A, ENO1, ERP29, ETFA, F12, F13A1, FABP4, FABP5, FASN, FBLN1, FBLN5, FERMT2, FKBP10, FKBP1A, FLNB, FMOD, FSCN1, GAPDH, GBP6, GDI1, GLIPR2, GLUL, GNB2, GOT1, GPD1, GPI, GPX1, GPX3, GSN, GSS, GSTA4, H2BC21, HADHA, HADHB, HBA1, HBB, HBE1, HCRT, HMGN1, HNRNPC, HNRNPU, HP, HPX, HSP90AA1, HSP90AB1, HSP90B1, HSPA1A, HSPA1B, HSPA5, HSPA6, HSPA8, HSPB1, HSPD1, HSPG2, IDH1, IFNG, IGFALS, IGHA1, IGHA2, IGHG1, IGHG3, IGHG4, IGHM, IGHV3-23, IGHV4OR15-8, IGKC, IGLC1, IGLV1-44, IGLV1-51, IGLV2-23, IGLV3-1, IQGAP1, ITGA7, ITGB1, ITIH4, KARS1, KCTD12, KIF5B, KNG1, KRT1, KRT5, LAMA2, LAMA5, LAMB1, LAMB2, LAMC1, LAMP2, LANCL1, LGALS1, LMNA, LPL, LYZ, MAOA, MCAM, MELTF, MFAP4, MGLL, MIF, MMP2, MSN, MYH9, MYO1C, MYO1E, NCF2, NCL, NID1, NPEPPS, ODC1, OGN, OLFML1, OLFML3, P4HB, PARK7, PDIA3, PDIA4, PEBP1, PGK1, PIP, PKM, PLG, PLIN1, POSTN, PPIA, PPIB, PRDX1, PRDX2, PRDX3, PRDX4, PRDX6, PRELP, PRXL2A, PSMA1, PSMA2, PSMA6, PSMA7, PSMB2, PSMB4, PSMB5, PTBP1, PTGES3, PTX3, PYROXD1, RAB10, RAB14, RAB1A, RAB39A, RAB7A, RAC1, RAN, RAP1A, RAP1B, RASSF5, RHOA, RPL11, RPS3, RPSA, RSU1, S100A11, S100A4, SERPINA1, SERPINC1, SERPIND1, SERPINF1, SERPINH1, SFN, SLC27A1, SLPI, SOD1, SOD2, SORBS1, SRI, SYNCRIP, TAPBP, TF, TGFBI, THBS4, THY1, TIMP2, TLN1, TMEM109, TMX1, TNC, TNXB, TPT1, TRIB2, TUBA1B, TUBB, TUFM, UBA1, UBB, UBE2K, UCHL1, UFC1, VCL, VCP, VIM, VPS35, VWF, YWHAB, YWHAG, YWHAZ

response to endogenous stimulus	0.001	Group24	A2M, ACAT1, ACSL1, ACTA1, ACTA2, ACTB, ADIPOQ, AGT, ANXA1, APOA1, APOA2, ATP1A1, ATP5F1A, CA2, CACNA2D1, CALR, CD36, CD81, CD9, CDH13, CLEC3B, CLU, COL1A1, COL1A2, COL6A1, CTSD, DCN, EEF1A1, EHD1, FERMT2, FKBP1A, FMOD, GOT1, GPD1, GPI, GPX1, HADHA, HNRNPC, HNRNPU, HSP90AA1, HSP90AB1, HSP90B1, HSPA1A, HSPA1B, HSPA5, HSPA8, HSPB1, IDH1, IGHA2, IQGAP1, ITGB1, LPL, MMP2, MYO1C, MYO1E, NCL, PARK7, PKM, POSTN, PTGES3, RAB10, RAB14, RAN, RAP1A, RAP1B, RHOA, SERPINF1, SLC27A1, SORBS1, TIMP2, TNC, TNXB, VIM, VPS35, YWHAG
response to chemical	0.000	Group24	A2M, ACAT1, ACSL1, ACTA1, ACTB, ACTG1, ACTN4, ADIPOQ, AKR1A1, ALB, ALDH1A1, ALKBH1, ANXA1, AOC3, APOA1, APOA2, APOA4, APOD, ATP1A1, ATP5F1A, ATP5F1B, C2CD2L, C5, CA2, CACNA2D1, CALR, CAMK2D, CANX, CBR1, CD36, CD81, CD9, CDC42, CDH13, CLEC3B, CLU, CNRIP1, COL18A1, COL1A1, COL1A2, COL6A1, COL6A3, CPB2, CRYAB, CTSD, CTSG, DPYSL3, EIF5A, ENO1, F12, FABP4, FASN, FBLN5, FLNB, GAPDH, GBP6, GDI1, GLUL, GOT1, GPD1, GPI, GPX1, GPX3, GSN, GSS, GSTA4, HADHA, HADHB, HBA1, HBB, HBE1, HNRNPC, HNRNPU, HP, HPX, HSP90AA1, HSP90AB1, HSP90B1, HSPA1A, HSPA1B, HSPA5, HSPA6, HSPA8, HSPB1, HSPD1, HSPG2, IDH1, IFNG, IGHA2, IQGAP1, ITGB1, ITIH4, KIF5B, LAMA2, LAMB2, LAMC1, LANCL1, LMNA, LPL, MELTF, MIF, MMP2, MSN, MYO1C, MYO1E, NCL, NPEPPS, P4HB, PARK7, PDIA3, PGK1, PIP, PKM, POSTN, PPIA, PPIB, PRDX1, PRDX2, PRDX3, PRDX4, PRDX6, PRXL2A, PSMB2, PTGES3, PYROXD1, RAB10, RAC1, RAN, RAP1A, RAP1B, RHOA, RPS3, RPSA, S100A4, SERPIND1, SERPINF1, SERPINH1, SLC27A1, SLPI, SOD1, SOD2, SORBS1, SRI, SYNCRIP, TF, THBS4, TIMP2, TNC, TUBA1B, TUFM, UBE2K, UCHL1, VCP, VIM, VPS35, YWHAG

response to organic substance	0.000	Group24	A2M, ACAT1, ACSL1, ACTA1, ACTB, ACTG1, ACTN4, ADIPOQ, AKR1A1, ALDH1A1, ANXA1, APOA1, APOA2, APOA4, ATP1A1, ATP5F1A, ATP5F1B, C2CD2L, CA2, CACNA2D1, CALR, CAMK2D, CANX, CD36, CD81, CD9, CDC42, CDH13, CLEC3B, CLU, CNRIP1, COL18A1, COL1A1, COL1A2, COL6A1, COL6A3, CPB2, CRYAB, CTSD, CTSG, DPYSL3, EIF5A, F12, FABP4, FASN, FLNB, GAPDH, GBP6, GDI1, GLUL, GOT1, GPD1, GPI, GPX1, GPX3, GSN, HADHA, HADHB, HBA1, HBB, HBE1, HNRNPC, HNRNPU, HP, HPX, HSP90AA1, HSP90AB1, HSP90B1, HSPA1A, HSPA1B, HSPA5, HSPA6, HSPA8, HSPB1, HSPD1, IDH1, IFNG, IGHA2, IQGAP1, ITGB1, ITIH4, KIF5B, LPL, MIF, MMP2, MSN, MYO1C, MYO1E, NCL, P4HB, PARK7, PDIA3, PKM, POSTN, PRDX2, PRDX3, PSMB2, PTGES3, RAB10, RAN, RAP1A, RAP1B, RHOA, RPS3, RPSA, SERPINF1, SERPINH1, SLC27A1, SLPI, SOD1, SOD2, SORBS1, SRI, SYNCRIP, THBS4, TIMP2, TNC, TUBA1B, TUFM, UBE2K, VCP, VIM, VPS35, YWHAG
response to inorganic substance	0.001	Group24	ANXA1, APOA4, ATP5F1A, CALR, CAMK2D, CD36, CLU, CNRIP1, COL18A1, COL1A1, CRYAB, FABP4, FBLN5, GDI1, GPI, GPX1, GSS, HBA1, HBB, HP, HSP90B1, IQGAP1, MELTF, MMP2, PARK7, PRDX1, PRDX2, PRDX3, RPS3, SERPINF1, SOD1, SOD2, TF, VCP
cellular response to chemical stimulus	0.000	Group24	ACSL1, ACTA1, ACTB, ACTG1, ACTN4, ADIPOQ, AKR1A1, ALB, ALDH1A1, ANXA1, APOA1, APOA4, ATP1A1, ATP5F1A, ATP5F1B, C2CD2L, C5, CA2, CACNA2D1, CALR, CAMK2D, CBR1, CD36, CDC42, CLEC3B, CLU, CNRIP1, COL1A1, COL1A2, COL6A1, CPB2, CTSD, CTSG, DPYSL3, EIF5A, ENO1, FABP4, FASN, FBLN5, FLNB, GAPDH, GBP6, GOT1, GPD1, GPX1, GPX3, GSN, GSTA4, HADHB, HBA1, HBB, HBE1, HNRNPU, HP, HPX, HSP90AB1, HSP90B1, HSPA1A, HSPA1B, HSPA5, HSPA8, HSPB1, HSPD1, IFNG, IQGAP1, KIF5B, LANCL1, LMNA, LPL, MELTF, MIF, MMP2, MSN, MYO1C, MYO1E, NCL, NPEPPS, P4HB, PARK7, PDIA3,

			PGK1, PKM, POSTN, PPIA, PPIB, PRDX1, PRDX2, PRDX3, PRDX4, PRDX6, PRXL2A, PTGES3, PYROXD1, RAB10, RAC1, RAN, RAP1A, RAP1B, RHOA, RPS3, RPSA, SERPINF1, SLC27A1, SOD1, SOD2, SORBS1, SRI, SYNCRIP, TF, THBS4, TNC, TUBA1B, UBE2K, UCHL1, VCP, VIM, VPS35, YWHAG
response to oxygen-containing compound	0.000	Group24	A2M, ACSL1, ACTB, ADIPOQ, AKR1A1, ALDH1A1, ANXA1, APOA2, APOA4, APOD, ATP1A1, ATP5F1A, C2CD2L, CA2, CACNA2D1, CALR, CAMK2D, CD36, CLU, COL18A1, COL1A1, COL1A2, COL6A1, COL6A3, CPB2, CRYAB, CTSD, CTSG, FABP4, FBLN5, GDI1, GLUL, GOT1, GPD1, GPI, GPX1, GPX3, HADHA, HADHB, HBA1, HBB, HNRNPC, HP, HSP90AA1, HSP90B1, IGHA2, IQGAP1, LPL, MIF, MMP2, MSN, MYO1C, MYO1E, NCL, PARK7, PKM, POSTN, PRDX1, PRDX2, PRDX3, RAB10, RAP1A, RAP1B, RHOA, RPS3, RPSA, SERPINF1, SLC27A1, SLPI, SOD1, SOD2, SORBS1, SRI, TNC, TUFM, VCP, VIM, VPS35, YWHAG
NADH regeneration	0.022	Group25	ENO1, PGAM1, PGK1, PGK2, PKM, TPI1
small molecule metabolic process	0.000	Group25	ABHD5, ACAT1, ACO2, ACSL1, ACSS2, ADIPOQ, AKR1A1, ALDH1A1, ALDH2, ALDOA, ANXA1, APOA1, APOA2, APOA4, APOB, APOD, ATP5F1A, ATP5F1B, ATP5IF1, ATP5MF, ATP5PB, ATP5PO, C3, CA1, CA2, CBR1, CD36, CFH, CMPK1, CRMP1, CYB5R3, DDAH2, DLD, DPYSL2, DPYSL3, ECHS1, ENO1, ETFA, FABP5, FASN, FUT2, GAPDH, GC, GLO1, GLUD1, GLUL, GOT1, GPD1, GPD2, GPI, GPX1, GSS, HADHA, HADHB, HSPA1A, HSPA1B, HSPA8, HYI, IDH1, IFNG, ILVBL, INPP5J, KARS1, LDHA, LDHB, LPL, MDH1, MDH2, MGLL, MIF, ODC1, PARK7, PGAM1, PGK1, PGK2, PKM, PTGES3, RAN, SDHA, SDHB, SLC27A1, SLPI, SOD1, SORBS1, SUCLA2, TALDO1, TKT, TNXB, TPI1, UGDH, VCP
organic acid metabolic process	0.000	Group25	ABHD5, ACAT1, ACO2, ACSL1, ACSS2, ADIPOQ, AKR1A1, ALDH1A1, ALDOA, ANXA1, APOA4, C3, CBR1, CD36, CFH, DDAH2, DLD, ECHS1, ENO1, ETFA, FABP5, FASN, GAPDH, GLUD1, GLUL, GOT1, GPD1, GPI, GPX1, GSS, HADHA,

			HADHB, HYI, IDH1, IFNG, ILVBL, KARS1, LDHA, LDHB, LPL, MDH1, MDH2, MGLL, MIF, ODC1, PARK7, PGAM1, PGK1, PGK2, PKM, PTGES3, SDHA, SLC27A1, SUCLA2, TNXB, TPI1, UGDH
generation of precursor metabolites and energy	0.000	Group25	ACAT1, ACO2, ACSS2, ADIPOQ, ALDH2, ALDOA, ATP5F1A, ATP5F1B, ATP5IF1, ATP5MF, ATP5PB, ATP5PO, CFH, DLD, ENO1, ENOX2, ETFA, GAPDH, GBE1, GPD1, GPD2, GPI, IDH1, IFNG, LDHA, MDH1, MDH2, NCF2, NIPSNAP2, PARK7, PGAM1, PGK1, PGK2, PKM, PTGES3, RHOA, SDHA, SDHB, SOD2, SORBS1, SUCLA2, TALDO1, TKT, TPI1, VCP
small molecule biosynthetic process	0.011	Group25	ACSS2, ADIPOQ, AKR1A1, ALDH1A1, ANXA1, APOA1, APOA4, APOB, CBR1, CYB5R3, ENO1, FABP5, FASN, GLUD1, GLUL, GOT1, GPD1, GPI, IFNG, ILVBL, LPL, MDH1, MDH2, MGLL, MIF, PARK7, PGAM1, PGK1, PGK2, PTGES3, SOD1, SUCLA2, TPI1, UGDH
monosaccharide metabolic process	0.017	Group25	ADIPOQ, AKR1A1, ALDOA, APOD, ENO1, FABP5, FUT2, GAPDH, GOT1, GPD1, GPI, MDH1, MDH2, PGAM1, PGK1, PGK2, PKM, SLPI, SORBS1, TPI1
nucleobase-containing small molecule metabolic process	0.003	Group25	ACAT1, ACSL1, ACSS2, ALDOA, ATP5F1A, ATP5F1B, ATP5IF1, ATP5MF, ATP5PB, ATP5PO, CMPK1, CRMP1, DLD, DPYSL2, DPYSL3, ENO1, GPD1, HSPA1A, HSPA1B, HSPA8, IDH1, KARS1, LDHB, MDH1, MDH2, PARK7, PGAM1, RAN, SDHA, SDHB, SLPI, SUCLA2, TALDO1, TKT, UGDH, VCP
canonical glycolysis	0.022	Group25	ENO1, PGAM1, PGK1, PGK2, PKM, TPI1
detoxification	0.000	Group26	AKR1A1, ALB, ALDH1A1, APOA4, CD36, FBLN5, GPX1, GPX3, HBA1, HBB, HBE1, HP, LANCL1, PARK7, PRDX1, PRDX2, PRDX3, PRDX4, PRDX6, PRXL2A, SOD1, SOD2
response to oxidative stress	0.000	Group26	ADIPOQ, ANXA1, APOA4, APOD, CD36, CLU, COL1A1, CRYAB, FBLN5, GPX1, GPX3, GSS, HBA1, HBB, HP, HSPA1A, HSPA1B, IDH1, KRT1, MELTF, MMP2, PARK7, PPIA, PRDX1, PRDX2, PRDX3, PRDX4, PRDX6, PSMB5, PYROXD1, RPS3, SOD1, SOD2

response to toxic substance	0.000	Group26	AKR1A1, ALB, ALDH1A1, APOA4, CD36, FBLN5, GPX1, GPX3, HBA1, HBB, HBE1, HP, LANCL1, PARK7, PRDX1, PRDX2, PRDX3, PRDX4, PRDX6, PRXL2A, SOD1, SOD2
response to inorganic substance	0.001	Group26	ANXA1, APOA4, ATP5F1A, CALR, CAMK2D, CD36, CLU, CNRIP1, COL18A1, COL1A1, CRYAB, FABP4, FBLN5, GDI1, GPI, GPX1, GSS, HBA1, HBB, HP, HSP90B1, IQGAP1, MELTF, MMP2, PARK7, PRDX1, PRDX2, PRDX3, RPS3, SERPINF1, SOD1, SOD2, TF, VCP
reactive oxygen species metabolic process	0.000	Group26	AGT, APOA4, ARF4, ATP5IF1, CBR1, CD36, CRYAB, CSRP1, FBLN5, GPX1, GPX3, HBA1, HBB, HBE1, HP, NCF2, PARK7, PRDX1, PRDX2, PRDX3, PRDX4, RAC1, RHOA, SOD1, SOD2
response to oxygen-containing compound	0.000	Group26	A2M, ACSL1, ACTB, ADIPOQ, AKR1A1, ALDH1A1, ANXA1, APOA2, APOA4, APOD, ATP1A1, ATP5F1A, C2CD2L, CA2, CACNA2D1, CALR, CAMK2D, CD36, CLU, COL18A1, COL1A1, COL1A2, COL6A1, COL6A3, CPB2, CRYAB, CTSD, CTSG, FABP4, FBLN5, GDI1, GLUL, GOT1, GPD1, GPI, GPX1, GPX3, HADHA, HADHB, HBA1, HBB, HNRNPC, HP, HSP90AA1, HSP90B1, IGHA2, IQGAP1, LPL, MIF, MMP2, MSN, MYO1C, MYO1E, NCL, PARK7, PKM, POSTN, PRDX1, PRDX2, PRDX3, RAB10, RAP1A, RAP1B, RHOA, RPS3, RPSA, SERPINF1, SLC27A1, SLPI, SOD1, SOD2, SORBS1, SRI, TNC, TUFM, VCP, VIM, VPS35, YWHAG
hydrogen peroxide metabolic process	0.000	Group26	APOA4, GPX1, GPX3, HBA1, HBB, HBE1, HP, PARK7, PRDX1, PRDX2, PRDX3, PRDX4, RAC1, SOD1, SOD2
cellular detoxification	0.000	Group26	AKR1A1, ALB, ALDH1A1, APOA4, CD36, FBLN5, GPX1, GPX3, HBA1, HBB, HBE1, HP, LANCL1, PARK7, PRDX1, PRDX2, PRDX3, PRDX4, PRDX6, PRXL2A, SOD1, SOD2
cellular oxidant detoxification	0.000	Group26	ALB, APOA4, CD36, FBLN5, GPX1, GPX3, HBA1, HBB, HBE1, HP, PARK7, PRDX1, PRDX2, PRDX3, PRDX4, PRDX6, PRXL2A, SOD1, SOD2

immune system process	0.000	Group27	A2M, ACTB, ACTG1, ADIPOQ, ANXA1, APOA1, APOA2, APOA4, APOD, ATP5IF1, C3, C4A, C4BPA, C5, C6, C7, C8A, C9, CALR, CD151, CD36, CD59, CD81, CD9, CDC42, CFB, CFH, CFI, CLU, CNRIP1, CSRP1, CTSD, CTSG, F12, FASN, FKBP1A, FLNB, GAPDH, GBP6, GPI, GPX1, GSN, H2BC21, HNRNPC, HP, HPX, HSP90AA1, HSPA1A, HSPA1B, HSPA9, HSPD1, IFNG, IGHA1, IGHA2, IGHG1, IGHG3, IGHG4, IGHM, IGHV3-23, IGHV4OR15-8, IGKC, IGLC1, IGLV1-44, IGLV1-51, IGLV2-23, IGLV3-1, ITGA7, ITGB1, KARS1, KIF5B, KRT1, LGALS1, LYZ, MIF, MMP2, MSN, MYH9, MYO1C, MYO1E, NCF2, PARK7, PDIA3, PIP, PPIA, PPIB, PRDX1, PRDX2, PRXL2A, PSMA1, PSMA7, PSMB4, PSMB9, PSME1, PTX3, RAB10, RAB6A, RAC1, RASSF5, RHOA, RPS3, RPSA, SAR1B, SCIN, SLPI, SOD1, SYNCRIP, TAPBP, TF, THBS4, THY1, TUBB, UBE2K, VIM, YWHAZ
biological process involved in interspecies interaction between organisms	0.000	Group27	A2M, ACTA2, ACTG1, ADIPOQ, ANXA1, APOA4, APOB, C3, C4A, C4BPA, C5, C6, C7, C8A, C9, CALR, CCT5, CD36, CD81, CDC42, CFB, CFH, CFI, CFL1, CLU, COTL1, CSRP1, CTSG, EEF1A1, EIF5A, ENO1, F12, FABP4, FASN, FLNB, GAPDH, GBP6, GPX1, GSN, H2BC21, HADHB, HNRNPC, HP, HPX, HSP90AA1, HSP90AB1, HSPA1A, HSPA1B, HSPA8, HSPB1, HSPD1, IFNG, IGHA1, IGHA2, IGHG1, IGHG3, IGHG4, IGHM, ITGB1, KIF5B, KRT1, LGALS1, LPL, LYZ, MIF, MYO1C, MYO1E, NCF2, ODC1, P4HB, PLG, PPIA, PPIB, PRDX1, PRDX2, PRDX3, PSMA2, PSMA7, PTX3, RAB14, RAB1A, RAB7A, RHOA, RPSA, SLPI, SOD2, SYNCRIP, TF, TPT1, TUBB, UBE2K, VIM, YWHAZ
response to stimulus	0.000	Group27	A2M, ACAT1, ACO2, ACSL1, ACTA1, ACTA2, ACTB, ACTG1, ACTN4, ADIPOQ, AGT, AKR1A1, ALB, ALDH1A1, ALKBH1, ANXA1, ANXA2, AOC3, APOA1, APOA2, APOA4, APOB, APOD, ARHGDIA, ARHGDIB, ATP1A1, ATP5F1A, ATP5F1B, ATP5IF1, BRAP, C2CD2L, C3, C4A, C4BPA, C5, C6, C7, C8A, C9, CA2, CABP1, CACNA2D1, CALR, CAMK2D, CANX, CAP1, CBR1, CCT5, CD151, CD163, CD36, CD59, CD81, CD9,

			CDC42, CDH13, CFB, CFH, CFI, CFL1, CIRBP, CLEC3B, CLU, CNRIP1, COL15A1, COL18A1, COL1A1, COL1A2, COL6A1, COL6A3, COTL1, CPB2, CRYAB, CSRP1, CTSD, CTSG, DCN, DDAH2, DPYSL2, DPYSL3, EEF1A1, EHD1, EIF5A, ENO1, ERP29, ETFA, F12, F13A1, FABP4, FABP5, FASN, FBLN1, FBLN5, FERMT2, FKBP10, FKBP1A, FLNB, FMOD, FSCN1, GAPDH, GBP6, GDI1, GLIPR2, GLUL, GNB2, GOT1, GPD1, GPI, GPX1, GPX3, GSN, GSS, GSTA4, H2BC21, HADHA, HADHB, HBA1, HBB, HBE1, HCRT, HMGN1, HNRNPC, HNRNPU, HP, HPX, HSP90AA1, HSP90AB1, HSP90B1, HSPA1A, HSPA1B, HSPA5, HSPA6, HSPA8, HSPB1, HSPD1, HSPG2, IDH1, IFNG, IGFALS, IGHA1, IGHA2, IGHG1, IGHG3, IGHG4, IGHM, IGHV3-23, IGHV4OR15-8, IGKC, IGLC1, IGLV1-44, IGLV1-51, IGLV2-23, IGLV3-1, IQGAP1, ITGA7, ITGB1, ITIH4, KARS1, KCTD12, KIF5B, KNG1, KRT1, KRT5, LAMA2, LAMA5, LAMB1, LAMB2, LAMC1, LAMP2, LANCL1, LGALS1, LMNA, LPL, LYZ, MAOA, MCAM, MELTF, MFAP4, MGLL, MIF, MMP2, MSN, MYH9, MYO1C, MYO1E, NCF2, NCL, NID1, NPEPPS, ODC1, OGN, OLFML1, OLFML3, P4HB, PARK7, PDIA3, PDIA4, PEBP1, PGK1, PIP, PKM, PLG, PLIN1, POSTN, PPIA, PPIB, PRDX1, PRDX2, PRDX3, PRDX4, PRDX6, PRELP, PRXL2A, PSMA1, PSMA2, PSMA6, PSMA7, PSMB2, PSMB4, PSMB5, PTBP1, PTGES3, PTX3, PYROXD1, RAB10, RAB14, RAB1A, RAB39A, RAB7A, RAC1, RAN, RAP1A, RAP1B, RASSF5, RHOA, RPL11, RPS3, RPSA, RSU1, S100A11, S100A4, SERPINA1, SERPINC1, SERPIND1, SERPINF1, SERPINH1, SFN, SLC27A1, SLPI, SOD1, SOD2, SORBS1, SRI, SYNCRIP, TAPBP, TF, TGFBI, THBS4, THY1, TIMP2, TLN1, TMEM109, TMX1, TNC, TNXB, TPT1, TRIB2, TUBA1B, TUBB, TUFM, UBA1, UBB, UBE2K, UCHL1, UFC1, VCL, VCP, VIM, VPS35, VWF, YWHAB, YWHAG, YWHAZ
response to stress	0.000	Group27	A2M, ACAT1, ACO2, ACTB, ACTG1, ADIPOQ, AGT, ALB, ALKBH1, ANXA1, ANXA2, AOC3, APOA1, APOA4, APOD, ATP5IF1, C3, C4A, C4BPA, C5, C6, C7, C8A, C9, CALR, CANX,

			CD151, CD163, CD36, CD59, CD81, CD9, CDC42, CFB, CFH, CFI, CIRBP, CLU, CNRIP1, COL18A1, COL1A1, COTL1, CPB2, CRYAB, CSRP1, CTSG, DPYSL3, ENO1, ERP29, F12, F13A1, FABP4, FASN, FBLN1, FBLN5, FERMT2, FKBP10, FLNB, FSCN1, GAPDH, GBP6, GLUL, GPI, GPX1, GPX3, GSN, GSS, H2BC21, HBA1, HBB, HCRT, HMGN1, HNRNPC, HP, HPX, HSP90AA1, HSP90AB1, HSP90B1, HSPA1A, HSPA1B, HSPA5, HSPA6, HSPA8, HSPB1, HSPD1, HSPG2, IDH1, IFNG, IGHA1, IGHA2, IGHG1, IGHG3, IGHG4, IGHM, ITGB1, ITIH4, KARS1, KIF5B, KNG1, KRT1, LAMB2, LAMC1, LAMP2, LGALS1, LMNA, LPL, LYZ, MCAM, MELTF, MGLL, MIF, MMP2, MYH9, MYO1C, MYO1E, NCF2, NPEPPS, P4HB, PARK7, PDIA3, PDIA4, PGK1, PLG, PLIN1, POSTN, PPIA, PRDX1, PRDX2, PRDX3, PRDX4, PRDX6, PRELP, PSMA1, PSMA6, PSMA7, PSMB4, PSMB5, PTX3, PYROXD1, RAB14, RAB1A, RAC1, RHOA, RPS3, RPSA, SERPINA1, SERPINC1, SERPIND1, SERPINH1, SFN, SLC27A1, SLPI, SOD1, SOD2, SYNCRIP, TF, THBS4, THY1, TLN1, TMEM109, TMX1, TNC, TNXB, TPT1, TUBB, UBA1, UBE2K, UCHL1, UFC1, VCL, VCP, VIM, VPS35, VWF, YWHAZ
response to external stimulus	0.000	Group27	A2M, ACAT1, ACSL1, ACTA1, ACTA2, ACTG1, ADIPOQ, AGT, ALB, ALKBH1, ANXA1, ANXA2, APOA1, APOA4, APOB, C3, C4A, C4BPA, C5, C6, C7, C8A, C9, CALR, CCT5, CD36, CD81, CD9, CDC42, CDH13, CFB, CFH, CFI, CFL1, CLU, COL1A1, COTL1, CPB2, CSRP1, CTSG, EIF5A, ENO1, F12, FABP4, FASN, FLNB, GAPDH, GBP6, GLUL, GPI, GPX1, GSN, H2BC21, HADHB, HCRT, HNRNPC, HP, HPX, HSP90AA1, HSPA1A, HSPA1B, HSPA5, HSPA8, HSPB1, HSPD1, IFNG, IGHA1, IGHA2, IGHG1, IGHG3, IGHG4, IGHM, KARS1, KIF5B, KNG1, KRT1, KRT5, LAMA2, LAMB2, LAMC1, LAMP2, LGALS1, LPL, LYZ, MGLL, MIF, MMP2, MYO1C, MYO1E, NCF2, ODC1, PARK7, PLG, POSTN, PPIA, PPIB, PRDX1, PRDX2, PRDX3, PSMA1, PSMA2, PSMA6, PSMA7, PSMB4, PTX3, RAB14, RAB1A, RAB7A, RAC1, RHOA, RPSA,

			S100A4, SERPINC1, SERPIND1, SLPI, SOD1, SOD2, SYNCRIP, TF, THBS4, TNC, TPT1, TUBB, UBE2K, VIM, VPS35, YWHAZ
response to biotic stimulus	0.000	Group27	A2M, ACTA2, ACTG1, ADIPOQ, ANXA1, APOA4, APOB, C3, C4A, C4BPA, C5, C6, C7, C8A, C9, CALR, CCT5, CD36, CDC42, CFB, CFH, CFI, CFL1, CLU, COTL1, CSRP1, CTSG, EIF5A, ENO1, F12, FABP4, FLNB, GAPDH, GBP6, GPX1, GSN, H2BC21, HADHB, HNRNPC, HP, HPX, HSP90AA1, HSPA1A, HSPA1B, HSPA5, HSPB1, HSPD1, IFNG, IGHA1, IGHA2, IGHG1, IGHG3, IGHG4, IGHM, KIF5B, KRT1, LPL, LYZ, MIF, MYO1C, MYO1E, NCF2, ODC1, PRDX1, PRDX2, PRDX3, PSMA2, PSMA7, PTX3, RAB14, RAB1A, RAB7A, RHOA, RPSA, SLPI, SOD2, SYNCRIP, TF, TPT1, TUBB, UBE2K, VIM, YWHAZ
regulation of response to stimulus	0.024	Group27	A2M, ACTA2, ACTB, ACTG1, ACTN4, ADIPOQ, AGT, ANXA1, ANXA2, APOA1, APOD, ARHGDIA, ARHGDIB, BRAP, C3, C4A, C4BPA, C5, C6, C7, C8A, C9, CALR, CAMK2D, CD36, CD59, CD81, CD9, CDC42, CDH13, CFB, CFH, CFI, CLU, CNRIP1, COL1A1, CPB2, CTSD, CTSG, DCN, EIF5A, ENO1, ERP29, ETFA, F12, FABP4, FABP5, FASN, FBLN1, FBLN5, FERMT2, FKBP1A, FLNB, GAPDH, GLIPR2, GPI, GPX1, HNRNPC, HPX, HSP90AA1, HSP90AB1, HSPA1A, HSPA1B, HSPA5, HSPB1, HSPD1, IFNG, IGHA1, IGHA2, IGHG1, IGHG3, IGHG4, IGHM, IGKC, IGLC1, IQGAP1, ITGB1, KARS1, KCTD12, KNG1, KRT1, LAMA2, LAMB1, LAMB2, LAMC1, LGALS1, LMNA, LPL, MAOA, MGLL, MIF, MYO1C, MYO1E, NCL, NID1, P4HB, PARK7, PDIA3, PEBP1, PLG, POSTN, PPIA, PRDX1, PRDX2, PSMA1, PSMA6, PSMA7, PSMB4, PTBP1, RAB7A, RAC1, RAP1A, RAP1B, RHOA, RPL11, RPS3, RPSA, S100A4, SERPINC1, SOD1, SOD2, SORBS1, THBS4, THY1, TNC, TNXB, TPT1, UBB, UBE2K, UCHL1, VCP, VPS35, VWF, YWHAB, YWHAG, YWHAZ
defense response	0.000	Group27	A2M, ACTG1, ADIPOQ, AGT, ANXA1, AOC3, APOA1, APOA4, APOD, C3, C4A, C4BPA, C5, C6, C7, C8A, C9, CD163, CD36, CD81, CDC42, CFB, CFH, CFI, CLU, COTL1,

			CSRP1, CTSG, F12, FABP4, FASN, FLNB, GAPDH, GBP6, GPX1, GSN, H2BC21, HNRNPC, HP, HPX, HSP90AA1, HSPA1A, HSPA1B, HSPD1, HSPG2, IFNG, IGHA1, IGHA2, IGHG1, IGHG3, IGHG4, IGHM, ITGB1, ITIH4, KARS1, KIF5B, KNG1, KRT1, LGALS1, LPL, LYZ, MGLL, MIF, MYO1C, MYO1E, NCF2, PARK7, PRDX1, PRDX2, PSMA1, PSMA6, PSMA7, PSMB4, PTX3, RAB14, RAB1A, RAC1, RPSA, SERPINA1, SLPI, SOD1, SYNCRIP, TF, TNC, TUBB, UBE2K, VIM, VPS35, YWHAZ
response to external biotic stimulus	0.000	Group27	A2M, ACTA2, ACTG1, ADIPOQ, ANXA1, APOA4, APOB, C3, C4A, C4BPA, C5, C6, C7, C8A, C9, CALR, CCT5, CD36, CDC42, CFB, CFH, CFI, CFL1, CLU, COTL1, CSRP1, CTSG, EIF5A, ENO1, F12, FABP4, FLNB, GAPDH, GBP6, GPX1, GSN, H2BC21, HADHB, HNRNPC, HP, HPX, HSP90AA1, HSPA1A, HSPA1B, HSPB1, HSPD1, IFNG, IGHA1, IGHA2, IGHG1, IGHG3, IGHG4, IGHM, KIF5B, KRT1, LPL, LYZ, MIF, MYO1C, MYO1E, NCF2, ODC1, PRDX1, PRDX2, PRDX3, PSMA2, PSMA7, PTX3, RAB14, RAB1A, RAB7A, RHOA, RPSA, SLPI, SOD2, SYNCRIP, TF, TPT1, TUBB, UBE2K, VIM, YWHAZ
response to other organism	0.000	Group27	A2M, ACTA2, ACTG1, ADIPOQ, ANXA1, APOA4, APOB, C3, C4A, C4BPA, C5, C6, C7, C8A, C9, CALR, CCT5, CD36, CDC42, CFB, CFH, CFI, CFL1, CLU, COTL1, CSRP1, CTSG, EIF5A, ENO1, F12, FABP4, FLNB, GAPDH, GBP6, GPX1, GSN, H2BC21, HADHB, HNRNPC, HP, HPX, HSP90AA1, HSPA1A, HSPA1B, HSPB1, HSPD1, IFNG, IGHA1, IGHA2, IGHG1, IGHG3, IGHG4, IGHM, KIF5B, KRT1, LPL, LYZ, MIF, MYO1C, MYO1E, NCF2, ODC1, PRDX1, PRDX2, PRDX3, PSMA2, PSMA7, PTX3, RAB14, RAB1A, RAB7A, RHOA, RPSA, SLPI, SOD2, SYNCRIP, TF, TPT1, TUBB, UBE2K, VIM, YWHAZ
cell adhesion	0.000	Group28	ACTB, ACTG1, ACTN4, ADIPOQ, ANXA1, ANXA2, AOC3, APOA1, APOA4, APOD, ATP5F1B, CALR, CD151, CD36, CD81, CD9, CDC42, CDH13, COL12A1, COL14A1, COL15A1,

			COL18A1, COL1A1, COL6A1, COL6A3, CSRP1, CTSG, FBLN1, FBLN5, FERMT2, FUT2, HBB, HSPB1, HSPD1, HSPG2, IFNG, IGFALS, ITGA7, ITGB1, KNG1, LAMA2, LAMA4, LAMA5, LAMB1, LAMB2, LAMC1, LGALS1, LGALS7, LPP, MCAM, MELTF, MFAP4, MMP2, MSN, MYH9, NID1, NID2, P4HB, PDIA3, PLG, POSTN, PPIA, PRDX2, RAB10, RAB1A, RAC1, RHOA, RPS3, RPSA, RSU1, S100A11, SORBS1, SPON1, TGFBI, THBS4, THY1, TLN1, TLN2, TNC, TNXB, TUBB, VCL, VWF
coagulation	0.000	Group28	ACTB, ACTG1, ANXA2, CD36, CD59, CD9, CPB2, CSRP1, CTSG, F12, F13A1, FBLN1, HBB, HSPB1, KNG1, KRT1, MYH9, PDIA3, PLG, PPIA, PRDX2, SERPINA1, SERPINC1, SERPIND1, TLN1, TUBB, VCL, VWF
response to wounding	0.000	Group28	ACTB, ACTG1, ANXA1, ANXA2, APOA4, APOD, CD151, CD36, CD59, CD9, CPB2, CSRP1, CTSG, DPYSL3, F12, F13A1, FBLN1, FERMT2, FKBP10, GPX1, HBB, HSPB1, ITGB1, KNG1, KRT1, LAMB2, LAMC1, MCAM, MMP2, MYH9, PDIA3, PLG, PPIA, PRDX2, RAC1, RHOA, SERPINA1, SERPINC1, SERPIND1, SOD1, SOD2, TLN1, TNC, TUBB, VCL, VWF
regulation of body fluid levels	0.000	Group28	ACTB, ACTG1, ANXA2, CD36, CD59, CD9, CPB2, CSRP1, CTSG, F12, F13A1, FBLN1, FLNB, GPI, HBB, HSPB1, KNG1, KRT1, MYH9, PDIA3, PLG, PPIA, PRDX2, SERPINA1, SERPINC1, SERPIND1, TLN1, TUBB, VCL, VWF
regulation of coagulation	0.013	Group28	ANXA2, CD36, CD9, CPB2, F12, KNG1, KRT1, PLG, PRDX2, SERPINC1
regulation of hemostasis	0.008	Group28	ANXA2, CD36, CD9, CPB2, F12, KNG1, KRT1, PLG, PRDX2, SERPINC1
blood coagulation	0.000	Group28	ACTB, ACTG1, ANXA2, CD36, CD59, CD9, CPB2, CSRP1, CTSG, F12, F13A1, FBLN1, HBB, HSPB1, KNG1, KRT1, MYH9, PDIA3, PLG, PPIA, PRDX2, SERPINA1, SERPINC1, SERPIND1, TLN1, TUBB, VCL, VWF
platelet activation	0.005	Group28	ACTB, ACTG1, CD9, CSRP1, CTSG, HBB, HSPB1, MYH9, PDIA3, PPIA, TLN1, TUBB, VCL, VWF

positive regulation of response to wounding	0.039	Group28	ACTG1, ANXA1, CD36, CPB2, F12, FERMT2, ITGB1, PLG, PRDX2
regulation of blood coagulation	0.006	Group28	ANXA2, CD36, CD9, CPB2, F12, KNG1, KRT1, PLG, PRDX2, SERPINC1
macromolecule localization	0.013	Group29	ABHD5, ACSL1, ACTB, ACTG1, ACTN4, ADIPOQ, AFM, AGT, ANXA1, ANXA2, APOA1, APOA2, APOA4, APOB, APOD, ARF4, ARHGDIA, ARL6IP1, ATP5IF1, C2CD2L, C3, CABP1, CALR, CANX, CAVIN1, CCT3, CCT5, CCT7, CD36, CD81, CDC42, CLU, COL1A1, COPG1, CSRP1, CTSD, EHD1, EHD2, ERP29, ESYT1, FABP4, FABP5, FBLN5, FERMT2, FKBP1A, GDI1, GLUD1, GLUL, GSN, HADHA, HNRNPA2B1, HNRNPU, HSP90AA1, HSP90AB1, HSP90B1, HSPA5, HSPA8, HSPA9, HSPB1, HSPD1, IFNG, ITGB1, KIF5B, KRT5, LAMA5, LAMP2, LMNA, LPL, MIF, MSN, MYH9, MYO1C, MYO1E, NPEPPS, OBSL1, PARK7, PDIA4, PPIA, RAB10, RAB11A, RAB14, RAB1A, RAB1B, RAB2A, RAB39A, RAB3D, RAB5C, RAB6A, RAB7A, RAN, RAP1A, RASSF5, RPL11, RPSA, SAR1B, SCIN, SELENBP1, SFN, SLC27A1, SORBS1, SRI, TCP1, THY1, VCL, VCP, VPS35, YWHAB, YWHAG, YWHAZ
establishment of localization	0.023	Group29	ACSL1, ACTB, ACTG1, ACTN4, ADIPOQ, AFM, AGT, ANXA1, ANXA2, APOA1, APOA2, APOA4, APOB, APOD, ARF4, ARHGDIA, ARL6IP1, ATP1A1, ATP5F1A, ATP5F1B, ATP5IF1, ATP5MF, ATP5PB, ATP5PO, C2CD2L, C3, C4A, C4BPA, CA2, CABP1, CACNA2D1, CALR, CAMK2D, CANX, CAP1, CAVIN1, CCT3, CCT5, CCT7, CD151, CD163, CD36, CD81, CD9, CDC42, CDH13, CFH, CFI, CFL1, CLU, COL1A1, COPG1, CP, CPB2, CRYAB, CSRP1, CTSD, CYB5RL, DPYSL2, EHD1, EHD2, ERP29, ESYT1, FABP4, FABP5, FBLN5, FKBP1A, FLNB, FTL, GC, GDI1, GLUD1, GNB2, GSN, HADHA, HBA1, HBB, HBE1, HCRT, HNRNPA2B1, HNRNPC, HNRNPU, HPX, HSP90AA1, HSP90B1, HSPA1A, HSPA5, HSPA8, HSPA9, HSPB1, HSPD1,

			HSPG2, IFNG, ITGB1, KIF5B, LAMP2, LMNA, MELTF, MIF, MRC2, MSN, MYH9, MYO1C, MYO1E, NCF2, NIPSNAP2, NPEPPS, P4HB, PARK7, PDIA4, PFN2, PPIA, PTX3, RAB10, RAB11A, RAB14, RAB1A, RAB1B, RAB2A, RAB39A, RAB3D, RAB5C, RAB6A, RAB7A, RAC1, RAN, RAP1A, RAP1B, RPL11, RPSA, SAR1B, SCIN, SELENBP1, SERPINA7, SFN, SLC27A1, SOD1, SORBS1, SRI, TAPBP, TCP1, TF, THY1, TMEM109, TPT1, TUBA1B, TUBA1C, TUBB, UBB, UCHL1, VCP, VPS35, YWHAB, YWHAG, YWHAZ
regulation of localization	0.000	Group29	ABHD5, ACSL1, ACTB, ACTG1, ACTN4, ADIPOQ, AGT, ANXA1, ANXA2, APOA1, APOA2, APOA4, APOB, APOD, ARHGDIA, ARL6IP1, ATP1A1, ATP5IF1, C2CD2L, C3, C4A, CA2, CABP1, CACNA2D1, CALR, CAMK2D, CCT3, CCT5, CCT7, CD151, CD36, CD81, CDC42, CDH13, CFH, CLU, CPB2, CRYAB, CSRP1, CTSD, EHD1, EHD2, ERP29, FABP5, FERMT2, FKBP1A, FLNB, GDI1, GLUD1, GLUL, GNB2, GSN, HADHA, HCRT, HNRNPC, HNRNPU, HSP90AA1, HSP90AB1, HSPA1A, HSPA8, IFNG, ITGB1, KIF5B, KRT5, LMNA, LPL, MIF, MSN, MYO1C, MYO1E, NIPSNAP2, NPEPPS, PARK7, PFN2, PPIA, PTX3, RAB11A, RAB14, RAB3D, RAB7A, RAC1, RAN, RAP1A, RAP1B, RASSF5, SAR1B, SCIN, SFN, SOD1, SORBS1, SRI, TCP1, TF, THY1, TMEM109, VCL, VCP, VPS35, YWHAZ
vesicle-mediated transport	0.014	Group29	ACTB, ACTG1, ADIPOQ, ANXA1, ANXA2, APOA1, APOA2, ARF4, ARHGDIA, C3, C4A, C4BPA, CALR, CANX, CAP1, CD151, CD163, CD36, CD81, CD9, CDC42, CDH13, CFI, CLU, COPG1, CSRP1, DPYSL2, EHD1, EHD2, GDI1, GSN, HNRNPC, HSPA8, HSPG2, IFNG, ITGB1, MRC2, MSN, MYH9, MYO1E, NCF2, P4HB, PFN2, PTX3, RAB10, RAB11A, RAB14, RAB1A, RAB1B, RAB2A, RAB39A, RAB3D, RAB5C, RAB6A, RAB7A, RAC1, RAP1A, RAP1B, RPSA, SAR1B, SCIN, SOD1, TAPBP, TF, VCP, VPS35
regulation of transport	0.000	Group29	ACSL1, ACTB, ACTG1, ACTN4, ADIPOQ, AGT, ANXA1, ANXA2, APOA1, APOA2, APOA4, APOD, ARHGDIA,

			ARL6IP1, ATP1A1, ATP5IF1, C2CD2L, C3, C4A, CA2, CABP1, CACNA2D1, CALR, CAMK2D, CD151, CD36, CD81, CDC42, CDH13, CFH, CLU, CPB2, CRYAB, EHD1, EHD2, ERP29, FABP5, FKBP1A, FLNB, GDI1, GLUD1, GNB2, HADHA, HCRT, HNRNPC, HSP90AA1, HSPA1A, HSPA8, IFNG, ITGB1, KIF5B, MIF, MSN, MYO1C, MYO1E, NIPSNAP2, NPEPPS, PARK7, PFN2, PPIA, PTX3, RAB11A, RAB3D, RAB7A, RAC1, RAN, RAP1A, RAP1B, SAR1B, SCIN, SFN, SOD1, SORBS1, SRI, TF, THY1, TMEM109, VPS35
positive regulation of transport	0.000	Group29	ACSL1, ACTB, ACTN4, ADIPOQ, ANXA1, ANXA2, APOA1, APOA2, ARL6IP1, ATP5IF1, C2CD2L, C3, C4A, CA2, CACNA2D1, CALR, CD151, CD36, CD81, CDC42, CFH, CLU, CPB2, EHD1, EHD2, FLNB, GLUD1, HCRT, HNRNPC, HSP90AA1, IFNG, ITGB1, KIF5B, MIF, MSN, MYO1C, MYO1E, NIPSNAP2, NPEPPS, PARK7, PPIA, PTX3, RAB3D, RAB7A, RAN, SAR1B, SCIN, SFN, SOD1, SORBS1, SRI, TF, THY1, VPS35
regulation of cellular component organization	0.000	Group29	ACTB, ACTG1, ADIPOQ, AEBP1, AGT, ANXA1, ANXA2, APOA1, APOA2, APOA4, APOD, ARF4, ARL6IP1, ATP5IF1, C3, C4A, CALR, CAMK2D, CAND1, CCT3, CCT5, CCT7, CD151, CD36, CDC42, CDH13, CFH, CFL1, CLU, COTL1, CPB2, CRMP1, CRYAB, DCN, DPYSL2, DPYSL3, EHD1, EHD2, EIF5A, ENO1, FERMT2, FSCN1, GDI1, GPX1, GSN, HNRNPA2B1, HNRNPC, HNRNPU, HSP90AA1, HSPA1A, HSPA1B, HSPA5, HSPA8, IFNG, INPP5J, ITGB1, LAMA2, LAMB1, LAMB2, LAMC1, LMNA, MELTF, MSN, MYH9, MYO1C, MYO1E, NID1, OBSL1, PARK7, PFN1, PFN2, PTX3, RAB1B, RAB7A, RAC1, RAP1A, RAP1B, RHOA, RPS3, SAR1B, SCIN, SERPINF1, SFN, SOD1, SPTAN1, SPTB, TCP1, TF, THY1, TLN1, TNC, TNXB, TUBB, TUBB4A, VAT1, VCL, VCP, VIM, VPS35, YWHAZ
regulation of cellular localization	0.000	Group29	ACTB, ADIPOQ, ANXA2, APOD, ARHGDIA, ATP5IF1, C2CD2L, CABP1, CAMK2D, CCT3, CCT5, CCT7, CD36, CD81, CRYAB, CTSD, EHD1, EHD2, ERP29, FERMT2, FKBP1A,

			GDI1, GLUD1, GLUL, GSN, HADHA, HSP90AA1, HSP90AB1, HSPA8, IFNG, ITGB1, KIF5B, KRT5, LMNA, MSN, MYO1C, MYO1E, NPEPPS, PARK7, PPIA, RAB14, RAN, RAP1A, RASSF5, SAR1B, SFN, SORBS1, SRI, TCP1, VCL, VCP, VPS35, YWHAZ
endocytosis	0.000	Group29	ACTB, ACTG1, ADIPOQ, ANXA1, ANXA2, APOA1, APOA2, C3, C4A, C4BPA, CALR, CANX, CAP1, CD151, CD163, CD36, CD81, CD9, CDC42, CDH13, CFI, CLU, CSRP1, DPYSL2, EHD1, EHD2, GSN, HNRNPC, HSPG2, IFNG, ITGB1, MRC2, MYH9, MYO1E, NCF2, PTX3, RAB14, RAB1A, RAB39A, RAB5C, RAB7A, RAC1, RPSA, SOD1, TF
positive regulation of cellular component organization	0.001	Group29	AGT, ANXA1, ANXA2, APOA1, APOA2, ATP5IF1, C3, C4A, CALR, CAND1, CCT3, CCT5, CCT7, CD151, CD36, CDC42, CLU, CPB2, DCN, DPYSL3, EHD1, EHD2, EIF5A, FERMT2, FSCN1, GDI1, GPX1, GSN, HNRNPA2B1, HNRNPC, HSP90AA1, HSPA1A, HSPA1B, IFNG, MELTF, MSN, OBSL1, PARK7, PFN1, PFN2, PTX3, RAC1, RAP1A, RAP1B, RHOA, RPS3, SCIN, SERPINF1, SOD1, TCP1, TF, THY1, TNXB, VCP, VPS35
regulation of vesicle-mediated transport	0.006	Group29	ACTB, ACTG1, ADIPOQ, ANXA1, ANXA2, APOA1, APOA2, ARHGDIA, C3, C4A, CALR, CD151, CD36, CDC42, CDH13, CLU, EHD1, EHD2, HNRNPC, IFNG, MSN, PFN2, PTX3, RAB11A, RAB3D, RAB7A, RAC1, RAP1A, RAP1B, SOD1, TF
positive regulation of endocytosis	0.006	Group29	ANXA2, APOA1, APOA2, C3, C4A, CALR, CD151, CD36, CDC42, CLU, HNRNPC, IFNG, PTX3, SOD1, TF
locomotion	0.000	Group30	ACTA2, ACTG1, ACTN4, ADIPOQ, AGT, ALKBH1, ANXA1, APOA1, APOD, ARHGDIB, ATP5F1A, ATP5F1B, C5, CALR, CAVIN1, CD151, CD81, CD9, CDC42, CDH13, CLU, COL1A1, CTSG, DCN, DPYSL3, FBLN1, FERMT2, GLIPR2, GLUL, GPI, HSPA5, HSPB1, IFNG, ITGB1, KIF5B, KRT5, LAMA2, LAMA4, LAMA5, LAMB1, LAMB2, LAMC1, LMNA, MCAM, MIF, MMP2, MSN, MYO1C, MYO1E, PFN1, PFN2, PODN, POSTN, PPIA, PPIB, RAB11A, RAC1, RHOA, RPSA, S100A11,

			S100A4, SERPIND1, SERPINF1, SOD2, TF, THBS4, THY1, TNC, TNXB, VCL, VPS35
cell adhesion	0.000	Group30	ACTB, ACTG1, ACTN4, ADIPOQ, ANXA1, ANXA2, AOC3, APOA1, APOA4, APOD, ATP5F1B, CALR, CD151, CD36, CD81, CD9, CDC42, CDH13, COL12A1, COL14A1, COL15A1, COL18A1, COL1A1, COL6A1, COL6A3, CSRP1, CTSG, FBLN1, FBLN5, FERMT2, FUT2, HBB, HSPB1, HSPD1, HSPG2, IFNG, IGFALS, ITGA7, ITGB1, KNG1, LAMA2, LAMA4, LAMA5, LAMB1, LAMB2, LAMC1, LGALS1, LGALS7, LPP, MCAM, MELTF, MFAP4, MMP2, MSN, MYH9, NID1, NID2, P4HB, PDIA3, PLG, POSTN, PPIA, PRDX2, RAB10, RAB1A, RAC1, RHOA, RPS3, RPSA, RSU1, S100A11, SORBS1, SPON1, TGFBI, THBS4, THY1, TLN1, TLN2, TNC, TNXB, TUBB, VCL, VWF
cell motility	0.000	Group30	ACTA2, ACTB, ACTG1, ACTN4, ADIPOQ, AGT, ALKBH1, ANXA1, APOA1, APOB, APOD, ARF4, ARHGDIB, ATP5F1A, ATP5F1B, C5, CALR, CAP1, CAVIN1, CD151, CD81, CD9, CDC42, CDH13, CLU, COL1A1, CTSG, DCN, DPYSL3, FBLN1, FERMT2, FSCN1, GLIPR2, GLUL, GPI, GPX1, HSP90AA1, HSPA5, HSPB1, IFNG, IQGAP1, ITGA7, ITGB1, KRT2, KRT5, LAMA2, LAMA4, LAMA5, LAMB1, LAMC1, LMNA, MCAM, MIF, MMP2, MSN, MYH9, MYO1C, MYO1E, PFN1, PFN2, PGK2, PODN, POSTN, PPIA, PPIB, RAB11A, RAB1A, RAC1, RHOA, RPSA, S100A11, SERPINF1, SOD2, TF, THBS4, THY1, TNC, TNXB, VCL
tissue migration	0.000	Group30	ACTA1, ACTA2, AGT, ANXA1, APOA1, ATP5F1A, ATP5F1B, CALR, CDH13, DCN, GLIPR2, GLUL, GPI, GPX1, HSPB1, IFNG, ITGB1, KRT2, MYH9, PFN1, PFN2, RAB11A, RAC1, RHOA, SERPINF1
regulation of locomotion	0.000	Group30	ACTA2, ACTG1, ACTN4, ADIPOQ, AGT, ANXA1, APOD, ARHGDIB, ATP5F1A, ATP5F1B, C5, CALR, CAVIN1, CD151, CD81, CD9, CDC42, CDH13, COL1A1, DCN, DPYSL3, FBLN1, FERMT2, GLIPR2, GLUL, GPI, HSPA5, HSPB1, IFNG, ITGB1, KRT5, LAMA2, LAMA4, LAMA5, LAMB1, LMNA, MCAM,

			MIF, MMP2, MSN, MYO1C, MYO1E, PFN1, PFN2, PODN, POSTN, RAB11A, RAC1, RHOA, RPSA, S100A11, SERPINF1, SOD2, TF, THBS4, THY1, TNC, TNXB, VCL, VPS35
response to wounding	0.000	Group30	ACTB, ACTG1, ANXA1, ANXA2, APOA4, APOD, CD151, CD36, CD59, CD9, CPB2, CSRP1, CTSG, DPYSL3, F12, F13A1, FBLN1, FERMT2, FKBP10, GPX1, HBB, HSPB1, ITGB1, KNG1, KRT1, LAMB2, LAMC1, MCAM, MMP2, MYH9, PDIA3, PLG, PPIA, PRDX2, RAC1, RHOA, SERPINA1, SERPINC1, SERPIND1, SOD1, SOD2, TLN1, TNC, TUBB, VCL, VWF
cell migration	0.000	Group30	ACTA2, ACTG1, ACTN4, ADIPOQ, AGT, ALKBH1, ANXA1, APOA1, APOD, ARF4, ARHGDIB, ATP5F1A, ATP5F1B, C5, CALR, CAP1, CD151, CD81, CD9, CDC42, CDH13, CLU, COL1A1, CTSG, DCN, DPYSL3, FERMT2, FSCN1, GLIPR2, GLUL, GPI, GPX1, HSP90AA1, HSPA5, HSPB1, IFNG, IQGAP1, ITGA7, ITGB1, KRT2, KRT5, LAMA2, LAMA4, LAMA5, LAMB1, LAMC1, LMNA, MCAM, MIF, MMP2, MSN, MYH9, MYO1C, MYO1E, PFN1, PFN2, PODN, POSTN, PPIA, PPIB, RAB11A, RAB1A, RAC1, RHOA, RPSA, S100A11, SERPINF1, SOD2, THBS4, THY1, TNC, TNXB, VCL
cell-substrate adhesion	0.000	Group30	ACTG1, ACTN4, APOA1, APOD, CALR, CD36, CDC42, CDH13, COL1A1, FBLN1, FBLN5, FERMT2, ITGA7, ITGB1, LAMA5, LAMB1, LAMC1, MELTF, NID1, NID2, P4HB, PLG, POSTN, RAB1A, RAC1, RHOA, RSU1, SORBS1, THY1, TLN1, TNXB, VCL, VWF
positive regulation of locomotion	0.000	Group30	ACTA2, ACTG1, ACTN4, AGT, ANXA1, ATP5F1A, ATP5F1B, CALR, CAVIN1, CD151, CDC42, CDH13, COL1A1, FERMT2, GLIPR2, GPI, HSPA5, HSPB1, IFNG, ITGB1, LAMB1, MCAM, MMP2, MYO1C, MYO1E, PFN1, POSTN, RAB11A, RAC1, RHOA, RPSA, S100A11, SOD2, TF, THBS4, THY1, VPS35
epithelium migration	0.002	Group30	AGT, ANXA1, APOA1, ATP5F1A, ATP5F1B, CALR, CDH13, DCN, GLIPR2, GLUL, GPI, GPX1, HSPB1, IFNG, ITGB1, KRT2, MYH9, PFN1, PFN2, RAB11A, RAC1, RHOA, SERPINF1

regulation of cell adhesion	0.000	Group30	ACTB, ACTG1, ACTN4, ADIPOQ, ANXA1, APOA1, APOD, ATP5F1B, CALR, CD36, CD81, CD9, CDC42, CDH13, COL1A1, CTSG, FBLN1, FERMT2, FUT2, HSPD1, HSPG2, IFNG, KNG1, LAMA2, LAMA4, LAMA5, LAMB1, LAMB2, LAMC1, LGALS1, MELTF, MMP2, NID1, P4HB, PLG, POSTN, PRDX2, RAC1, RHOA, RPS3, RSU1, TGFBI, THY1, TLN1, TNC, TNXB, VCL
regulation of cell motility	0.000	Group30	ACTA2, ACTG1, ACTN4, ADIPOQ, AGT, ANXA1, APOD, ARHGDIB, ATP5F1A, ATP5F1B, C5, CALR, CAVIN1, CD151, CD81, CD9, CDC42, CDH13, COL1A1, DCN, DPYSL3, FBLN1, FERMT2, GLIPR2, GLUL, GPI, HSPA5, HSPB1, IFNG, ITGB1, KRT5, LAMA2, LAMA4, LAMA5, LAMB1, LMNA, MCAM, MIF, MMP2, MSN, MYO1C, MYO1E, PFN1, PFN2, PODN, POSTN, RAB11A, RAC1, RHOA, RPSA, S100A11, SERPINF1, SOD2, TF, THBS4, THY1, TNC, TNXB, VCL
negative regulation of cell adhesion	0.007	Group30	ACTN4, ADIPOQ, ANXA1, APOA1, APOD, ATP5F1B, CD9, CDH13, COL1A1, CTSG, FBLN1, HSPG2, KNG1, MELTF, MMP2, PLG, POSTN, PRDX2, RHOA, TGFBI, TNC
positive regulation of cell motility	0.000	Group30	ACTA2, ACTG1, ACTN4, AGT, ANXA1, ATP5F1A, ATP5F1B, CALR, CAVIN1, CD151, CDC42, CDH13, COL1A1, FERMT2, GLIPR2, GPI, HSPA5, HSPB1, IFNG, ITGB1, LAMB1, MCAM, MMP2, MYO1C, MYO1E, PFN1, POSTN, RAB11A, RAC1, RHOA, RPSA, S100A11, SOD2, TF, THBS4, THY1
regulation of epithelial cell migration	0.035	Group30	AGT, ANXA1, ATP5F1A, ATP5F1B, CALR, DCN, GLIPR2, GLUL, GPI, HSPB1, IFNG, PFN1, PFN2, RAB11A, RAC1, RHOA, SERPINF1
immune system process	0.000	Group31	A2M, ACTB, ACTG1, ADIPOQ, ANXA1, APOA1, APOA2, APOA4, APOD, ATP5IF1, C3, C4A, C4BPA, C5, C6, C7, C8A, C9, CALR, CD151, CD36, CD59, CD81, CD9, CDC42, CFB, CFH, CFI, CLU, CNRIP1, CSRP1, CTSD, CTSG, F12, FASN, FKBP1A, FLNB, GAPDH, GBP6, GPI, GPX1, GSN, H2BC21, HNRNPC, HP, HPX, HSP90AA1, HSPA1A, HSPA1B, HSPA9, HSPD1, IFNG, IGHA1, IGHA2, IGHG1, IGHG3, IGHG4, IGHM, IGHV3-23, IGHV4OR15-8, IGKC, IGLC1, IGLV1-44, IGLV1-

			51, IGLV2-23, IGLV3-1, ITGA7, ITGB1, KARS1, KIF5B, KRT1, LGALS1, LYZ, MIF, MMP2, MSN, MYH9, MYO1C, MYO1E, NCF2, PARK7, PDIA3, PIP, PPIA, PPIB, PRDX1, PRDX2, PRXL2A, PSMA1, PSMA7, PSMB4, PSMB9, PSME1, PTX3, RAB10, RAB6A, RAC1, RASSF5, RHOA, RPS3, RPSA, SAR1B, SCIN, SLPI, SOD1, SYNCRIP, TAPBP, TF, THBS4, THY1, TUBB, UBE2K, VIM, YWHAZ
biological process involved in interspecies interaction between organisms	0.000	Group31	A2M, ACTA2, ACTG1, ADIPOQ, ANXA1, APOA4, APOB, C3, C4A, C4BPA, C5, C6, C7, C8A, C9, CALR, CCT5, CD36, CD81, CDC42, CFB, CFH, CFI, CFL1, CLU, COTL1, CSRP1, CTSG, EEF1A1, EIF5A, ENO1, F12, FABP4, FASN, FLNB, GAPDH, GBP6, GPX1, GSN, H2BC21, HADHB, HNRNPC, HP, HPX, HSP90AA1, HSP90AB1, HSPA1A, HSPA1B, HSPA8, HSPB1, HSPD1, IFNG, IGHA1, IGHA2, IGHG1, IGHG3, IGHG4, IGHM, ITGB1, KIF5B, KRT1, LGALS1, LPL, LYZ, MIF, MYO1C, MYO1E, NCF2, ODC1, P4HB, PLG, PPIA, PPIB, PRDX1, PRDX2, PRDX3, PSMA2, PSMA7, PTX3, RAB14, RAB1A, RAB7A, RHOA, RPSA, SLPI, SOD2, SYNCRIP, TF, TPT1, TUBB, UBE2K, VIM, YWHAZ
cell killing	0.042	Group31	C3, C5, C6, C7, C8A, C9, CD59, CFH, CTSG, GAPDH, IFNG, IGHG1, KIF5B, LYZ, PRDX1, PSMA7, TUBB
immune effector process	0.000	Group31	A2M, ANXA1, APOA1, APOA2, C3, C4A, C4BPA, C5, C6, C7, C8A, C9, CD36, CD59, CD81, CFB, CFH, CFI, CLU, CSRP1, CTSG, GPI, HNRNPC, HPX, HSPD1, IFNG, IGHA1, IGHA2, IGHG1, IGHG3, IGHG4, IGHM, IGHV3-23, IGHV4OR15-8, IGKC, IGLC1, KARS1, KIF5B, KRT1, LGALS1, MIF, MYO1C, PRDX1, PRDX2, PSMA7, PTX3, RPSA, TUBB
response to stress	0.000	Group31	A2M, ACAT1, ACO2, ACTB, ACTG1, ADIPOQ, AGT, ALB, ALKBH1, ANXA1, ANXA2, AOC3, APOA1, APOA4, APOD, ATP5IF1, C3, C4A, C4BPA, C5, C6, C7, C8A, C9, CALR, CANX, CD151, CD163, CD36, CD59, CD81, CD9, CDC42, CFB, CFH, CFI, CIRBP, CLU, CNRIP1, COL18A1, COL1A1, COTL1, CPB2, CRYAB, CSRP1, CTSG, DPYSL3, ENO1, ERP29, F12, F13A1, FABP4, FASN, FBLN1, FBLN5, FERMT2, FKBP10, FLNB,

			FSCN1, GAPDH, GBP6, GLUL, GPI, GPX1, GPX3, GSN, GSS, H2BC21, HBA1, HBB, HCRT, HMGN1, HNRNPC, HP, HPX, HSP90AA1, HSP90AB1, HSP90B1, HSPA1A, HSPA1B, HSPA5, HSPA6, HSPA8, HSPB1, HSPD1, HSPG2, IDH1, IFNG, IGHA1, IGHA2, IGHG1, IGHG3, IGHG4, IGHM, ITGB1, ITIH4, KARS1, KIF5B, KNG1, KRT1, LAMB2, LAMC1, LAMP2, LGALS1, LMNA, LPL, LYZ, MCAM, MELTF, MGLL, MIF, MMP2, MYH9, MYO1C, MYO1E, NCF2, NPEPPS, P4HB, PARK7, PDIA3, PDIA4, PGK1, PLG, PLIN1, POSTN, PPIA, PRDX1, PRDX2, PRDX3, PRDX4, PRDX6, PRELP, PSMA1, PSMA6, PSMA7, PSMB4, PSMB5, PTX3, PYROXD1, RAB14, RAB1A, RAC1, RHOA, RPS3, RPSA, SERPINA1, SERPINC1, SERPIND1, SERPINH1, SFN, SLC27A1, SLPI, SOD1, SOD2, SYNCRIP, TF, THBS4, THY1, TLN1, TMEM109, TMX1, TNC, TNXB, TPT1, TUBB, UBA1, UBE2K, UCHL1, UFC1, VCL, VCP, VIM, VPS35, VWF, YWHAZ
immune response	0.003	Group31	A2M, ACTG1, ANXA1, APOA1, APOA2, APOA4, C3, C4A, C4BPA, C5, C6, C7, C8A, C9, CD36, CD59, CD81, CDC42, CFB, CFH, CFI, CLU, CNRIP1, CSRP1, CTSG, F12, FASN, FKBP1A, FLNB, GAPDH, GBP6, GPI, GPX1, GSN, H2BC21, HNRNPC, HPX, HSP90AA1, HSPA1A, HSPA1B, HSPD1, IFNG, IGHA1, IGHA2, IGHG1, IGHG3, IGHG4, IGHM, IGHV3-23, IGHV4OR15-8, IGKC, IGLC1, IGLV1-44, IGLV1-51, IGLV2-23, IGLV3-1, KARS1, KIF5B, KRT1, LGALS1, LYZ, MIF, MYO1C, MYO1E, NCF2, PARK7, PRDX1, PSMA1, PSMA7, PSMB4, PTX3, RPS3, RPSA, SLPI, SYNCRIP, TAPBP, TF, THY1, TUBB, UBE2K, VIM, YWHAZ
response to external stimulus	0.000	Group31	A2M, ACAT1, ACSL1, ACTA1, ACTA2, ACTG1, ADIPOQ, AGT, ALB, ALKBH1, ANXA1, ANXA2, APOA1, APOA4, APOB, C3, C4A, C4BPA, C5, C6, C7, C8A, C9, CALR, CCT5, CD36, CD81, CD9, CDC42, CDH13, CFB, CFH, CFI, CFL1, CLU, COL1A1, COTL1, CPB2, CSRP1, CTSG, EIF5A, ENO1, F12, FABP4, FASN, FLNB, GAPDH, GBP6, GLUL, GPI, GPX1, GSN, H2BC21, HADHB, HCRT, HNRNPC, HP, HPX, HSP90AA1,

			HSPA1A, HSPA1B, HSPA5, HSPA8, HSPB1, HSPD1, IFNG, IGHA1, IGHA2, IGHG1, IGHG3, IGHG4, IGHM, KARS1, KIF5B, KNG1, KRT1, KRT5, LAMA2, LAMB2, LAMC1, LAMP2, LGALS1, LPL, LYZ, MGLL, MIF, MMP2, MYO1C, MYO1E, NCF2, ODC1, PARK7, PLG, POSTN, PPIA, PPIB, PRDX1, PRDX2, PRDX3, PSMA1, PSMA2, PSMA6, PSMA7, PSMB4, PTX3, RAB14, RAB1A, RAB7A, RAC1, RHOA, RPSA, S100A4, SERPINC1, SERPIND1, SLPI, SOD1, SOD2, SYNCRIP, TF, THBS4, TNC, TPT1, TUBB, UBE2K, VIM, VPS35, YWHAZ
response to biotic stimulus	0.000	Group31	A2M, ACTA2, ACTG1, ADIPOQ, ANXA1, APOA4, APOB, C3, C4A, C4BPA, C5, C6, C7, C8A, C9, CALR, CCT5, CD36, CDC42, CFB, CFH, CFI, CFL1, CLU, COTL1, CSRP1, CTSG, EIF5A, ENO1, F12, FABP4, FLNB, GAPDH, GBP6, GPX1, GSN, H2BC21, HADHB, HNRNPC, HP, HPX, HSP90AA1, HSPA1A, HSPA1B, HSPA5, HSPB1, HSPD1, IFNG, IGHA1, IGHA2, IGHG1, IGHG3, IGHG4, IGHM, KIF5B, KRT1, LPL, LYZ, MIF, MYO1C, MYO1E, NCF2, ODC1, PRDX1, PRDX2, PRDX3, PSMA2, PSMA7, PTX3, RAB14, RAB1A, RAB7A, RHOA, RPSA, SLPI, SOD2, SYNCRIP, TF, TPT1, TUBB, UBE2K, VIM, YWHAZ
positive regulation of biological process	0.000	Group31	A2M, ABHD5, ACSL1, ACTA1, ACTA2, ACTB, ACTG1, ACTN4, ADIPOQ, AGT, ANXA1, ANXA2, APOA1, APOA2, APOA4, APOB, ARF4, ARL6IP1, ATP1A1, ATP5F1A, ATP5F1B, ATP5IF1, C2CD2L, C3, C4A, C4BPA, C5, C6, C7, C8A, C9, CA2, CACNA2D1, CALR, CAMK2D, CAND1, CAPNS1, CAVIN1, CBR1, CCT3, CCT5, CCT7, CD151, CD36, CD59, CD81, CDC42, CDH13, CFB, CFH, CFI, CFL1, CIRBP, CLEC3B, CLU, COL1A1, CPB2, CSRP1, CTSD, CTSG, DCN, DDAH2, DPYSL3, EEF2, EHD1, EHD2, EIF5A, ENO1, ERP29, ETFA, F12, FABP4, FABP5, FASN, FERMT2, FKBP1A, FLNB, FSCN1, GAPDH, GC, GDI1, GLIPR2, GLUD1, GPD1, GPI, GPX1, GSN, HADHA, HBB, HCRT, HMGN1, HNRNPA2B1, HNRNPC, HNRNPR, HNRNPU, HPX, HSP90AA1, HSP90AB1, HSPA1A, HSPA1B, HSPA5, HSPA8, HSPB1, HSPD1, HSPG2,

			IFNG, IGHA1, IGHA2, IGHG1, IGHG3, IGHG4, IGHM, IGKC, IGLC1, ILF2, IQGAP1, ITGB1, KARS1, KIF5B, KNG1, KRT1, KRT2, LAMA2, LAMB1, LAMB2, LAMC1, LGALS1, LMNA, LPL, LUM, MAOA, MCAM, MELTF, MIF, MMP2, MSN, MYH9, MYO1C, MYO1E, NCL, NID1, NIPSNAP2, NPEPPS, OBSL1, ODC1, P4HB, PAFAH1B2, PARK7, PDIA3, PFN1, PFN2, PIP, PKM, PLG, POSTN, PPIA, PPIB, PRDX2, PRDX3, PRDX6, PSMA7, PSME1, PTBP1, PTGES3, PTPA, PTX3, RAB11A, RAB1A, RAB1B, RAB3D, RAB7A, RAC1, RAN, RAP1A, RAP1B, RASSF5, RHOA, RPL11, RPS2, RPS3, RPSA, RSU1, S100A11, S100A4, SAR1B, SCIN, SERPINF1, SFN, SH3BGRL, SLC27A1, SOD1, SOD2, SORBS1, SPON1, SRI, SYNCRIP, TCP1, TF, THBS4, THRAP3, THY1, TNC, TNXB, TRIB2, UBB, UBE2K, VCP, VIM, VPS35, VWF
regulation of immune system process	0.001	Group31	A2M, ACTB, ADIPOQ, ANXA1, APOA1, APOA2, APOD, C3, C4A, C4BPA, C5, C6, C7, C8A, C9, CALR, CD36, CD59, CD81, CD9, CFB, CFH, CFI, CLU, CTSG, FKBP1A, GPI, GPX1, HNRNPC, HPX, HSP90AA1, HSPA1A, HSPA1B, HSPA9, HSPD1, IFNG, IGHA1, IGHA2, IGHG1, IGHG3, IGHG4, IGHM, IGKC, IGLC1, KARS1, KRT1, LGALS1, MIF, MSN, MYO1C, PARK7, PIP, PRDX2, PRXL2A, PSMA1, PSMA7, PSMB4, RAC1, RASSF5, RHOA, RPS3, RPSA, SCIN, SOD1, THBS4, THY1, UBE2K, YWHAZ
regulation of response to stimulus	0.024	Group31	A2M, ACTA2, ACTB, ACTG1, ACTN4, ADIPOQ, AGT, ANXA1, ANXA2, APOA1, APOD, ARHGDIA, ARHGDIB, BRAP, C3, C4A, C4BPA, C5, C6, C7, C8A, C9, CALR, CAMK2D, CD36, CD59, CD81, CD9, CDC42, CDH13, CFB, CFH, CFI, CLU, CNRIP1, COL1A1, CPB2, CTSD, CTSG, DCN, EIF5A, ENO1, ERP29, ETFA, F12, FABP4, FABP5, FASN, FBLN1, FBLN5, FERMT2, FKBP1A, FLNB, GAPDH, GLIPR2, GPI, GPX1, HNRNPC, HPX, HSP90AA1, HSP90AB1, HSPA1A, HSPA1B, HSPA5, HSPB1, HSPD1, IFNG, IGHA1, IGHA2, IGHG1, IGHG3, IGHG4, IGHM, IGKC, IGLC1, IQGAP1, ITGB1, KARS1, KCTD12, KNG1, KRT1, LAMA2, LAMB1, LAMB2, LAMC1,

			LGALS1, LMNA, LPL, MAOA, MGLL, MIF, MYO1C, MYO1E, NCL, NID1, P4HB, PARK7, PDIA3, PEBP1, PLG, POSTN, PPIA, PRDX1, PRDX2, PSMA1, PSMA6, PSMA7, PSMB4, PTBP1, RAB7A, RAC1, RAP1A, RAP1B, RHOA, RPL11, RPS3, RPSA, S100A4, SERPINC1, SOD1, SOD2, SORBS1, THBS4, THY1, TNC, TNXB, TPT1, UBB, UBE2K, UCHL1, VCP, VPS35, VWF, YWHAB, YWHAG, YWHAZ
leukocyte mediated immunity	0.006	Group31	C3, C4A, C4BPA, C5, C6, C7, C8A, C9, CD81, CFB, CFI, CLU, CTSG, HNRNPC, HPX, HSPD1, IGHA1, IGHA2, IGHG1, IGHG3, IGHG4, IGHM, IGHV3-23, IGHV4OR15-8, IGKC, IGLC1, KIF5B, PRDX1, PSMA7, TUBB
positive regulation of immune system process	0.008	Group31	A2M, ACTB, ANXA1, C3, C4A, C4BPA, C5, C6, C7, C8A, C9, CALR, CD36, CD59, CD81, CFB, CFH, CFI, CLU, CTSG, GPI, HNRNPC, HPX, HSP90AA1, HSPA1A, HSPA1B, HSPD1, IFNG, IGHA1, IGHA2, IGHG1, IGHG3, IGHG4, IGHM, IGKC, IGLC1, KARS1, KRT1, LGALS1, MIF, MYO1C, PARK7, PSMA7, RAC1, RHOA, RPS3, RPSA, THBS4, THY1, UBE2K
defense response	0.000	Group31	A2M, ACTG1, ADIPOQ, AGT, ANXA1, AOC3, APOA1, APOA4, APOD, C3, C4A, C4BPA, C5, C6, C7, C8A, C9, CD163, CD36, CD81, CDC42, CFB, CFH, CFI, CLU, COTL1, CSRP1, CTSG, F12, FABP4, FASN, FLNB, GAPDH, GBP6, GPX1, GSN, H2BC21, HNRNPC, HP, HPX, HSP90AA1, HSPA1A, HSPA1B, HSPD1, HSPG2, IFNG, IGHA1, IGHA2, IGHG1, IGHG3, IGHG4, IGHM, ITGB1, ITIH4, KARS1, KIF5B, KNG1, KRT1, LGALS1, LPL, LYZ, MGLL, MIF, MYO1C, MYO1E, NCF2, PARK7, PRDX1, PRDX2, PSMA1, PSMA6, PSMA7, PSMB4, PTX3, RAB14, RAB1A, RAC1, RPSA, SERPINA1, SLPI, SOD1, SYNCRIP, TF, TNC, TUBB, UBE2K, VIM, VPS35, YWHAZ
humoral immune response	0.000	Group31	A2M, C3, C4A, C4BPA, C5, C6, C7, C8A, C9, CD59, CD81, CFB, CFH, CFI, CLU, CTSG, GAPDH, GPI, H2BC21, HNRNPC, HPX, IFNG, IGHA1, IGHA2, IGHG1, IGHG3, IGHG4, IGHM, KRT1, LYZ, PSMA7, SLPI, TF

response to external biotic stimulus	0.000	Group31	A2M, ACTA2, ACTG1, ADIPOQ, ANXA1, APOA4, APOB, C3, C4A, C4BPA, C5, C6, C7, C8A, C9, CALR, CCT5, CD36, CDC42, CFB, CFH, CFI, CFL1, CLU, COTL1, CSRP1, CTSG, EIF5A, ENO1, F12, FABP4, FLNB, GAPDH, GBP6, GPX1, GSN, H2BC21, HADHB, HNRNPC, HP, HPX, HSP90AA1, HSPA1A, HSPA1B, HSPB1, HSPD1, IFNG, IGHA1, IGHA2, IGHG1, IGHG3, IGHG4, IGHM, KIF5B, KRT1, LPL, LYZ, MIF, MYO1C, MYO1E, NCF2, ODC1, PRDX1, PRDX2, PRDX3, PSMA2, PSMA7, PTX3, RAB14, RAB1A, RAB7A, RHOA, RPSA, SLPI, SOD2, SYNCRIP, TF, TPT1, TUBB, UBE2K, VIM, YWHAZ
positive regulation of response to stimulus	0.000	Group31	A2M, ACTA2, ACTB, ACTG1, ACTN4, ADIPOQ, AGT, ANXA1, APOA1, C3, C4A, C4BPA, C5, C6, C7, C8A, C9, CALR, CD36, CD59, CD81, CDC42, CDH13, CFB, CFH, CFI, CLU, COL1A1, CPB2, CTSD, CTSG, DCN, EIF5A, ERP29, ETFA, F12, FABP4, FABP5, FASN, FERMT2, FKBP1A, FLNB, GAPDH, GLIPR2, GPX1, HNRNPC, HPX, HSP90AA1, HSP90AB1, HSPA1A, HSPA1B, HSPB1, HSPD1, IFNG, IGHA1, IGHA2, IGHG1, IGHG3, IGHG4, IGHM, IGKC, IGLC1, IQGAP1, ITGB1, KARS1, KRT1, LAMA2, LAMB1, LAMB2, LAMC1, LGALS1, LPL, MAOA, MIF, MYO1C, MYO1E, NID1, PARK7, PDIA3, PLG, PPIA, PRDX2, PSMA7, PTBP1, RAC1, RAP1A, RAP1B, RHOA, RPL11, RPS3, RPSA, S100A4, SOD1, SORBS1, THBS4, THY1, TNXB, UBB, UBE2K, VCP, VPS35, VWF
response to other organism	0.000	Group31	A2M, ACTA2, ACTG1, ADIPOQ, ANXA1, APOA4, APOB, C3, C4A, C4BPA, C5, C6, C7, C8A, C9, CALR, CCT5, CD36, CDC42, CFB, CFH, CFI, CFL1, CLU, COTL1, CSRP1, CTSG, EIF5A, ENO1, F12, FABP4, FLNB, GAPDH, GBP6, GPX1, GSN, H2BC21, HADHB, HNRNPC, HP, HPX, HSP90AA1, HSPA1A, HSPA1B, HSPB1, HSPD1, IFNG, IGHA1, IGHA2, IGHG1, IGHG3, IGHG4, IGHM, KIF5B, KRT1, LPL, LYZ, MIF, MYO1C, MYO1E, NCF2, ODC1, PRDX1, PRDX2, PRDX3, PSMA2, PSMA7, PTX3, RAB14, RAB1A, RAB7A, RHOA,

			RPSA, SLPI, SOD2, SYNCRIP, TF, TPT1, TUBB, UBE2K, VIM, YWHAZ
regulation of response to external stimulus	0.020	Group31	A2M, ADIPOQ, AGT, ANXA1, ANXA2, APOA1, C3, C5, CALR, CD36, CD81, CD9, CDH13, CFH, CPB2, F12, FABP4, GPX1, HPX, HSP90AA1, HSPA1A, HSPA1B, HSPB1, HSPD1, IFNG, IGHA2, KARS1, KNG1, KRT1, LGALS1, LPL, MGLL, MIF, MYO1C, PARK7, PLG, PRDX2, PSMA1, PSMA6, PSMB4, RAC1, RPSA, SERPINC1, SOD1, THBS4, TNC, UBE2K, VPS35, YWHAZ
regulation of immune response	0.001	Group31	A2M, ANXA1, C3, C4A, C4BPA, C5, C6, C7, C8A, C9, CD36, CD59, CD81, CFB, CFH, CFI, CLU, CTSG, FKBP1A, GPX1, HNRNPC, HPX, HSP90AA1, HSPA1A, HSPA1B, HSPD1, IFNG, IGHA1, IGHA2, IGHG1, IGHG3, IGHG4, IGHM, IGKC, IGLC1, KARS1, KRT1, MYO1C, PARK7, PSMA1, PSMA7, PSMB4, RPS3, RPSA, THY1, UBE2K, YWHAZ
positive regulation of immune response	0.000	Group31	A2M, ANXA1, C3, C4A, C4BPA, C5, C6, C7, C8A, C9, CD36, CD59, CD81, CFB, CFH, CFI, CLU, CTSG, HNRNPC, HPX, HSP90AA1, HSPA1A, HSPA1B, HSPD1, IFNG, IGHA1, IGHA2, IGHG1, IGHG3, IGHG4, IGHM, IGKC, IGLC1, KARS1, KRT1, MYO1C, PARK7, PSMA7, RPS3, RPSA, THY1, UBE2K
defense response to other organism	0.000	Group31	A2M, ACTG1, ANXA1, APOA4, C3, C4A, C4BPA, C5, C6, C7, C8A, C9, CD36, CDC42, CFB, CFH, CFI, CLU, COTL1, CSRP1, CTSG, F12, FLNB, GAPDH, GBP6, GSN, H2BC21, HNRNPC, HP, HPX, HSP90AA1, HSPA1A, HSPA1B, HSPD1, IFNG, IGHA1, IGHA2, IGHG1, IGHG3, IGHG4, IGHM, KIF5B, KRT1, LYZ, MIF, MYO1C, MYO1E, NCF2, PRDX1, PSMA7, PTX3, RAB14, RAB1A, RPSA, SLPI, SYNCRIP, TF, TUBB, UBE2K, VIM, YWHAZ
activation of immune response	0.000	Group31	A2M, C3, C4A, C4BPA, C5, C6, C7, C8A, C9, CD36, CD59, CD81, CFB, CFH, CFI, CLU, HNRNPC, HSP90AA1, HSPA1A, HSPA1B, HSPD1, IGHA1, IGHA2, IGHG1, IGHG3, IGHG4, IGHM, IGKC, IGLC1, KRT1, MYO1C, PSMA7, RPS3, RPSA, THY1

innate immune response	0.000	Group31	A2M, ACTG1, ANXA1, APOA4, C3, C4A, C4BPA, C5, C6, C7, C8A, C9, CD36, CDC42, CFB, CFH, CFI, CLU, CSRP1, F12, FLNB, GAPDH, GBP6, GSN, H2BC21, HNRNPC, HPX, HSP90AA1, HSPA1A, HSPA1B, HSPD1, IFNG, IGHA2, IGHM, KIF5B, KRT1, MIF, MYO1C, MYO1E, NCF2, PRDX1, PSMA7, PTX3, RPSA, SLPI, SYNCRIP, TUBB, UBE2K, VIM, YWHAZ
complement activation	0.000	Group31	A2M, C3, C4A, C4BPA, C5, C6, C7, C8A, C9, CD59, CFB, CFH, CFI, CLU, HNRNPC, IGHA1, IGHA2, IGHG1, IGHG3, IGHG4, IGHM, KRT1, PSMA7
